# Rescue of *Sly* Expression Is Not Sufficient to Rescue Spermiogenic Phenotype of Mice with Deletions of Y Chromosome Long Arm

**DOI:** 10.3390/genes10020133

**Published:** 2019-02-12

**Authors:** Jonathan M. Riel, Yasuhiro Yamauchi, Victor A. Ruthig, Qushay U. Malinta, Mélina Blanco, Charlotte Moretti, Julie Cocquet, Monika A. Ward

**Affiliations:** 1 Institute for Biogenesis Research, John A. Burns School of Medicine, University of Hawaii, 1960 East-West Rd, Honolulu, HI 96822, USA; jriel@hawaii.edu (J.M.R.); yyamauch@hawaii.edu (Y.Y.); var13@duke.edu (V.A.R.); qushay20019@gmail.com (Q.U.M.); 2 INSERM, U1016, Institut Cochin, 75013 Paris, France; melina.blanco@inserm.fr (M.B.); charlotte.moretti@ens-lyon.fr (C.M.); julie.cocquet@inserm.fr (J.C.); 3 CNRS, UMR8104, 75014 Paris, France; 4 Université Paris Descartes, Sorbonne Paris Cité, Faculté de Médecine, 75014 Paris, France

**Keywords:** Y chromosome, testis, spermatogenesis, SLY, mouse, infertility, assisted reproduction

## Abstract

Mice with deletions of the Y-specific (non-PAR) region of the mouse Y chromosome long arm (NPYq) have sperm defects and fertility problems that increase proportionally to deletion size. Mice with abrogated function of NPYq-encoded gene *Sly* (sh367 Sly-KD) display a phenotype similar to that of NPYq deletion mutants but less severe. The milder phenotype can be due to insufficient *Sly* knockdown, involvement of another NPYq gene, or both. To address this question and to further elucidate the role of *Sly* in the infertile phenotype of mice with NPYq deletions, we developed an anti-SLY antibody specifically recognizing SLY1 and SLY2 protein isoforms and used it to characterize SLY expression in NPYq- and *Sly*-deficient mice. We also carried out transgene rescue by adding *Sly1/2* transgenes to mice with NPYq deletions. We demonstrated that SLY1/2 expression in mutant mice decreased proportionally to deletion size, with ~12% of SLY1/2 retained in shSLY sh367 testes. The addition of *Sly1/2* transgenes to mice with NPYq deletions rescued SLY1/2 expression but did not ameliorate fertility and testicular/spermiogenic defects. Together, the data suggest that *Sly* deficiency is not the sole underlying cause of the infertile phenotype of mice with NPYq deletions and imply the involvement of another NPYq gene.

## 1. Introduction

Deletions of the Y chromosome are frequently associated with spermatogenic defects both in mice and in humans. In mice, the male-specific, non-pairing Y chromosome long arm (NPYq), (also called MSYq for the male-specific region on the Y chromosome long arm), encompasses ~90% of the Y-specific DNA content and comprises mostly repetitive sequences including multiple copies of four distinct genes that are expressed in spermatids: *Ssty1*, *Ssty2* (Spermiogenesis specific transcript on the Y 1 and 2), *Sly* (Sycp3 like Y-linked), and *Srsy* (Serine rich, secreted, Y-linked) [1]. These multi-copy genes show a progressive reduction in transcript levels with increasing NPYq deficiency and are candidates for contributing to the sperm defects associated with NPYq deletions [2]. Mice with NPYq deletions have sperm defects and are sub- or infertile, with the severity of the phenotype increasing proportionally to the deletion size [3,4,5,6,7,8]. We succeeded in obtaining live offspring from the infertile males with NPYq deletions when intracytoplasmic sperm injection (ICSI) was used [8,9]; however, the low efficiency of assisted reproduction suggested that sperm impairment reached beyond their inability to transmit the paternal genome to the oocyte in vivo, and might have involved DNA changes. In support of this notion, we have subsequently shown that sperm from mice with severe NPYq deficiencies have DNA damage and abnormal chromatin packaging [10].

To assess which of the NPYq genes is responsible for the infertile phenotype associated with NPYq deficiency, we produced mice in which the function of NPYq-encoded gene *Sly* has been disrupted by transgenically-delivered short hairpin RNAs [11]. The characterization of these ‘shSLY mice’ (sh367 or Sly-KD for knocked down) revealed infertility, sperm headshape defects, and impairment in sperm chromatin packaging, as well as increased sperm DNA damage, similar to that noted in mice with severe NPYq deletions, but less severe [11,12]. These studies also revealed the underlying cause of Sly-KD and NPYq-spermiogenic phenotypes: Sly-KD or NPYq deletions were shown to lead to a de-repression of sex chromosome-encoded genes and to changes in sex chromatin structure in spermatids [11,13,14,15]. Molecular analyses showed that SLY1 protein directly regulates the expression of sex chromosome-encoded spermatid-expressed genes, as well as hundreds of spermatid-expressed autosomal genes, with many SLY1 target genes involved in transcriptional regulation and chromatin remodeling [11,14].

Yet, Sly-KD mice phenotype is milder than that of mice with a 9/10th or complete deletion of NPYq. This could be due to insufficient *Sly* knockdown in Sly-KD, involvement of another NPYq gene in the phenotype of mice with NPYq deficiency, or both. To address this question and to further elucidate the role of *Sly* in the infertile phenotype of mice with NPYq deletions, we undertook a two-pronged approach. First, if sperm abnormalities in NPYq-deficient mice are a consequence of *Sly* deficiency, then there should be a correlation between the extent of *Sly* reduction and the severity of sperm defects. We showed earlier that *Sly* transcript levels correlated well with the phenotype [16]. However, the analysis of SLY protein expression was hampered by the fact that the only available SLY antibody only detects the SLY protein long isoform, SLY1, and not the shorter SLY2. To overcome this problem, we developed a new anti-SLY1/2 antibody and used it to characterize SLY expression in NPYq- and *Sly*-deficient mice. Second, if sperm abnormalities in NPYq-deficient mice are a consequence of *Sly* deficiency, then transgenically bringing *Sly*/SLY expression in NPY deficient mice to normal levels, should rescue their infertile phenotype. To address this, we developed mice transgenic for *Sly* and placed the *Sly* transgene in the context of sub- and infertile NPYq-deficient genotypes.

We demonstrated first that Sly-KD mice retain limited quantities of SLY1 and 2 proteins. Importantly, we also showed that males with NPYq deficiency expressing transgenic SLY1 or SLY1/2 at levels comparable to wild-type males displayed fertility impairment and testicular/spermiogenic defects, suggesting the contribution of another NPYq gene to these phenotypes. 

## 2. Materials and Methods

### 2.1. Chemicals

Pregnant mares’ serum gonadotrophin (eCG) and human chorionic gonadotrophin (hCG) were purchased from Calbiochem (San Diego, CA, USA). All other chemicals were obtained from Sigma Chemical Co. (St Louis, MO, USA) unless otherwise stated.

### 2.2. Mice

Six-to-twelve week-old B6D2F1 (C57BL/6J × DBA/2) females (NCI, Raleigh, NC, USA) were used as oocyte donors for injections and CD-1 (Charles River, Wilmington, MA, USA) or Swiss Webster (NCI) mice were used as vasectomized males and surrogate/foster females for embryo transfer. The mice were fed ad libitum with a standard diet and maintained in a temperature- and light-controlled room (22 °C, 14 h light/10 h dark), in accordance with the guidelines of the Laboratory Animal Services at the University of Hawaii and guidelines presented in National Research Council’s (NCR) “Guide for Care and Use of Laboratory Animals” published by Institute for Laboratory Animal Research (ILAR) of the National Academy of Science, Bethesda, MD, 2011. The protocol for animal handling and treatment procedures was reviewed and approved by the Animal Care and Use Committee at the University of Hawaii (animal protocol number 06-010).

The mice of interest in this study were mice with NPYq and *Sly* deficiencies, described by us before [10,12], and mice with transgenic overexpression of *Sly* that were generated in this study. Mice with NPYq/*Sly* deficiencies were on a C57BL/6 genetic background. The XY^RIII^ males on the C57BL/6 background were used as wild-type controls. For a summary of investigated mice, see Table S1.

### 2.3. Production of Anti-*SLY* Antibody

To produce an anti-SLY1/2 antibody, a specific peptide (VKSPAFDKNENISPQ) identified by ClustalW alignment of proteins from the XLR family to which SLY belongs, was used to immunize mice using a standard approach. Polyclonal serum with the highest antibody titer was identified by ELISA and screened for specificity using dot blot. The serum that was SLY-specific and recognized both SLY1 and SLY2 isoforms was used to create hybridoma cell lines and transformed into monoclonal anti-SLY antibody. The antibody was further tested for specificity using dot blot, immunofluorescence with HEK293 cells transfected with *Sly1* and *Sly2* ORFs fused to FLAG tags, and Western blot with protein lysates from the testes from mice with NPYq and *Sly* deficiencies.

### 2.4. Production of *SLY1*, *SLY2*, *SLX*, and *SLXL1* Proteins

The open reading frames of *Sly1*, *Sly2*, *Slx*, and *Slxl1* were cloned in-frame with an N-terminal FLAG tag under the control of the CMV promoter of the pCDNA3.1 vector (Invitrogen, Carlsbad, CA, USA). HEK293T cells were transfected using X-treme Gene9 (Roche, Basel, Switzerland) according to the manufacturer’s instructions with a pCDNA3.1-CMV vector. Cells were collected for protein extraction 48 h after transfection and pelleted by centrifugation for 5 min at 1000 g. Cells were lysed using ice-cold lysis buffer (50mM Tris HCl with 150 mM NaCl pH 7.4, 1mM EDTA, 1% Triton X-100 and 1 x Complete, Mini, EDTA-free Protease Inhibitor Cocktail). Lysis solution was incubated with anti-FLAG affinity gel (Sigma A2220) at 4 °C overnight on a rotating platform. After centrifugation at 13,000× g at 4 °C for 5 min, the supernatant was discarded and purified protein was eluted from gel.

### 2.5. Dot-Blot

SLY1, SLY2, SLX, and fetal bovine serum (FBS) proteins were spot dropped on a nitrocellulose membrane and allowed to dry. The membrane was incubated in blocking buffer to prevent non-specific binding and then with an anti-SLY antibody followed by detection with anti-mouse HRP conjugated antibody (sc-2005; Santa Cruz, Dallas, TX, USA) at 1:5000 in PBST. After antibody binding and washes, the membrane was incubated in chemiluminescent solution (SuperSignal West Pico; Thermo Fisher Scientific, Waltham, MA, USA) and imaged by an ImageQuant LAS 4000 biomolecular imager (GE Healthcare Life Sciences, Chicago, IL, USA).

### 2.6. Immunofluorescence

Immunofluorescence on surface-spread HEK293T cells was performed as previously described [12]. Primary antibodies rabbit anti-FLAG (Sigma, SAB4301135) and SLY (this paper) were diluted 1/500 or used as hybridoma cell culture supernatant, respectively. Secondary antibodies anti-rabbit AF488 and anti-mouse AF488 (Life Technologies Invitrogen, R37118 and A-11001, respectively) were diluted at 1:500. Pictures were taken with an Olympus BX63 wide field fluorescent microscope (Olympus, Tokyo, Japan).

### 2.7. Western Blotting

Protein extraction and Western blot analyses were performed as described previously [12]. Briefly, 10 to 15 mg of testis or spermatid fraction protein extracts were run on a 12% sodium dodecyl sulfate (SDS)/polyacrylamide gel. Following transfer and blocking, membranes were incubated overnight with one of the following primary antibodies: anti-SLY1 [17], anti-SLY1/2 hybridoma cell culture supernatant, or anti-ACTB at 1:5000. Incubation with the corresponding secondary antibody coupled to peroxidase (anti-mouse IgG sc-2005 or anti-rabbit IgG sc-2313) and detection by chemiluminescence were carried out as described by the manufacturer (SuperSignal West Pico, Pierce Biotechnology, Rockford, IL, USA).

### 2.8. Production of Mice Transgenic for Sly

*Flag-Sly* transgenic mice were produced by pronuclear injection of a linearized construct containing either *Sly1* or *Sly2* reading frame, i.e., *Sly1* and *Sly2* isoforms fused with *Flag* sequence, under the control of the spermatid-specific promoter *SP10* (aka *Acrv1*). Fertilized oocytes were microinjected with the construct, using standard protocols. Transgenic founders carrying the *SP10-Flag-Sly1* or *SP10-Flag-Sly2* construct were identified by PCR (primers are shown in Table S2 [11,12,13,18,19]). The founders with germline transmission were used to propagate transgenic lines. The males from lines with significant *Sly* expression were used to obtain XX*Sly* transgenic females. These females were used for breeding with subfertile 2/3NPYq- males and as oocyte donors for ICSI with sperm from infertile 9/10NPYq- males. The resulting 2/3NPYq-*Sly* and 9/10NPYq-*Sly* males were characterized in respect to their fertility and spermatogenic phenotype. *Sly*-transgenic mice (no FLAG tag) were produced using the same strategy with the construct that did not have the *Flag* sequence.

### 2.9. Sperm Analyses

To analyze sperm number, motility and morphology, cauda epididymal sperm was released into HEPES-buffered CZB medium (HEPES-CZB [20]), and incubated for at least 10 min at 37 °C immediately before analysis. Sperm counts using a hemocytometer were the mean of three independent scorings per sample. For the analysis of sperm morphology, epididymal sperm were stained with silver nitrate as previously described [8]. Briefly, the sperm suspension (diluted as necessary with 0.9% NaCl) was smeared on three slides, allowed to dry, fixed in methanol and acetic acid (3:1), and stained with silver nitrate. The slides were coded and 100 sperm heads per slide were viewed at 1000× magnification and scored in blind fashion. Categorization of sperm head morphology was performed as previously described [8].

### 2.10. Assisted Reproduction

Female mice were induced to superovulate with the injection of 5 IU eCG and 5 IU hCG given 48 h apart. Oocyte collection and subsequent oocyte manipulation, including microinjections, were done in HEPES-CZB, with subsequent culture in CZB medium [21] in an atmosphere of 5% CO_2_ in air.

To obtain epididymal sperm, caudae epididymides were dissected and sperm were expressed with needles into HEPES-CZB or PBS or T6. Spermatozoa were allowed to disperse for 2–3 min at room temperature. The samples of epididymal cell suspension were used for analyses, for in vitro fertilization (IVF), or for ICSI.

For IVF, the epididymal sperm were capacitated in T6 medium [22] for 1.5 h at 37 °C in a humidified atmosphere of 5% CO_2_. In vitro fertilization was performed as previously described [23]. Gametes were co-incubated for 4 h. After co-incubation, the oocytes were washed several times with HEPES-CZB, followed by at least one wash with CZB. Embryos were cultured in CZB and observed at different time points for proper development: 24 h (2-cell stage), 48 h (4-or 8-cell stage), 72 h (morula or early blastocyst), and 96 h (blastocyst).

Intracytoplasmic sperm injection was carried out as previously described [24], within 1–2 h from oocyte and sperm collection. Sperm injected oocytes were transferred in CZB and cultured at 37 °C. The survival and activation of injected oocytes was scored 1–2 h and 6 h after the commencement of culture, respectively. The oocytes with two well-developed pronuclei and extruded second polar body were considered activated.

Embryos resulting from ICSI that reached the 2-cell stage were transferred to the oviducts (10–14 per oviduct) of CD1 females mated during the previous night with vasectomized CD1 males. Surrogate mothers were allowed to deliver and raise their offspring or had a cesarean section performed and the progeny raised by foster mothers. The progeny were genotyped after weaning (age, 21 days) and subsequently used for the analyses of fertility and spermiogenic phenotype.

### 2.11. Real-Time *RT-PCR*

For real-time reverse transcriptase polymerase chain reaction (RT-PCR), total testis RNA was extracted using Trizol and DNaseI treatment (Ambion, Austin, TX, USA), and purified using an RNeasy kit (Qiagen, Valencia, CA, USA). Reverse transcription of polyadenylated RNA was performed with Superscript Reverse Transcriptase IV, according to the manufacturer’s guidelines (Invitrogen). Real-time PCR was performed using SYBR Green PCR Master mix on an ABI QuantStudio 12K Flex machine (Applied Biosystems, Carlsbad, CA, USA). PCR reactions were incubated at 95 °C for 10 min followed by 35 PCR cycles (10 s at 95 °C and 60 s at 60 °C). For analysis of *Sly* expression, two types of PCR reactions were performed: (1) ‘*Sly1+2*’ amplifying both *Sly*_v1 and *Sly*_v2 transcripts [17] (primers *Sly* Global) and (2) ‘*Sly1*’ amplifying only *Sly*_v1 (primers *Sly* Long). All reactions were carried out in triplicate per assay and *Actb* was included as a loading control. The ∆Ct value for each individual sample was calculated by subtracting the average ∆Ct of *Actb* from the average ∆Ct of each tested gene. ∆∆Ct was calculated by subtracting the ∆Ct of each tested male from the average ∆Ct of the reference samples (non-transgenic siblings). The data were expressed as a fold value of expression level. Expression analysis was also done on several X and Y encoded transcripts to test for their upregulation in NPYq-deficient mice with and without the *Sly* transgene. Primer sequences are shown in supplementary material Table S2.

### 2.12. Development and Analyses of SP4 Transgenic Mice

An additional transgenic line expressing *Sly1* (*Flag-Sly1* SP4, subsequently called SP4) was developed and characterized independently in Cocquet lab in France. Overall, a similar approach and methodology were used as those described above for mice generated in Ward lab in Hawaii. The few differences are as follows:

Mice. All animals were on ~90% C57BL/6 background and processed at adult age (between 2- and 6-month-old males). The SP4 line was obtained by pronuclear micro-injection of a linearized construct containing a *Sly1* open reading frame fused with a *Flag* sequence, under the control of the spermatid-specific promoter *SP10* (aka *Acrv1*) [14]. Transgenic SP4 *Flag*-*Sly1* males with a wild-type Y chromosome (i.e., XY^RIII^ or XY^B10^) have already been described [14]. In brief, RT-qPCR and Western blot experiments showed a ~2-fold increase in *Sly1* transcript and SLY1 protein level in SP4 transgenic testes compared to testes from non-transgenic (WT) siblings. The 2/3NPYq- (2/3MSYq-) mice have a Y^RIII^ chromosome with a deletion removing approximately two-thirds of the NPYq. NPYq- (MSYq-) mice are X*Sxr*^a^Y*^X^ mice [3] and lack the entire Y-specific (non-PAR) gene content of NPYq, with the only Y-specific material provided by the Y short-arm-derived factor *Sxr^a^*. To produce SP4 transgenic males with a 2/3NPYq-, SP4 transgenic females were mated to 2/3NPYq- males. To produce SP4 transgenic males with NPYq deletion, SP4 transgenic females were mated to XY*Sxr^a^* males. Then SP4 transgenic XY*Sxr^a^* males were mated to XY*^X^ females to produce SP4 transgenic X*Sxr^a^*Y*^X^ males (i.e., SP4 transgenic NPYq- males) [25,26,27,28]. The animal procedures were in accordance with the United Kingdom Animal Scientific Procedures Act 1986 and subject to local ethical review in UK and France (Comite d’Ethique pour l’Experimentation Animale, Universite Paris Descartes; registration number CEEA34.JC.114.12).

Real-time quantitative PCR. For the quantification of *Flag*-*Sly* transgene expression, total RNA was extracted from adult testes and reverse-transcribed as described above. Real-time PCR was performed using Roche Light Cycler 480 and Bioline SensiFAST SYBR No-ROX Kit (Meridian Bioscience, Paris, France) using the primers described in Table S2. *Acrv1* was included on every plate as a loading control. *Acrv1* expression was not affected in mice with Y chromosome deletion or Sly-KD mice, checked by both microarray and qRT-PCR [11].

Western blot and immunofluorescence. Western blot and immunofluorescence analyses were performed using the same conditions as described before [29]. For Western blot analyses, anti-SLY1 antibody [17] was used at 1/3000; for immunofluorescence experiments, anti-SLY1 was used at 1/100. Alexa Fluor 594-conjugated peanut agglutinin lectin (Invitrogen) stains the developing acrosome and was used at 1/500 to stage testis tubules.

Analysis of sperm head morphology. Silver staining of sperm smears obtained from the initial caput epididymis was performed as described previously [7]. The analyses were performed in blind fashion. In comparison with classification of the Ward lab [8], the categories were as follows: slightly flattened = 1S + 2S, grossly flattened = 4G, and other gross abnormalities = 3G + 5G + 6G + 7G + 8G. 

### 2.13. Statistics

The transcript and protein expression, testis and body weight, and sperm number were analyzed with a *t*-test. Fertilization rate data were analyzed by Fisher’s Exact Test. Sperm headshape defects data were assessed by two-way ANOVA using the GraphPad Prism version 5.0 software after transforming all percentages to angles.

## 3. Results

### 3.1. New Anti-*SLY* Antibody Specifically Recognizes *SLY1* and *SLY2* Proteins

Thus far, analyses of SLY protein expression have been hampered by a lack of suitable antibody because the previously used serum recognized only one of the two existing SLY isoforms, SLY1. To overcome this limitation, we raised an antibody that specifically recognized both SLY1 and SLY2. 

A ClustalW alignment of XLR, SLX, SLX1, and SYCP3 with the predicted SLY1 and SLY2 amino acid sequences (Figure S1) identified a 15-amino acid, putatively SLY1- and SLY2-specific peptide. This peptide was used to generate a monoclonal anti-SLY1/2 antibody. The antibody was able to detect SLY1 and SLY2 proteins in HEK293 cells transfected with *Sly1* and *Sly2* ORF fused to the *Flag* tag but did not react with HEK293 cells transfected with *Flag*-*Slx* and *Slxl1* ORF (Figure S2). The antibody also detected SLY1 and SLY2, but not SLX, on dot blots (Figure S3).

**Figure 1:**
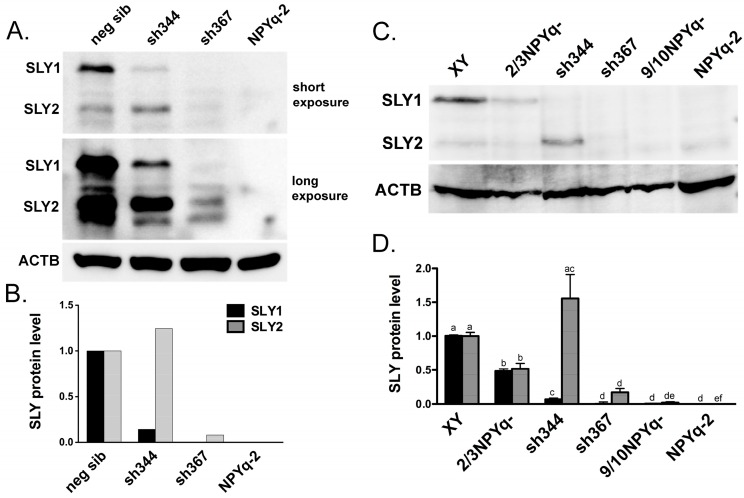
**SLY expression in males with NPYq- and *Sly*-specific deficiency**. (**A**,**B**) New anti-SLY antibody specifically recognizes SLY1 and SLY2 isoforms. (**A**) Exemplary Western blot detection of SLY1 (~40 kDa) and SLY2 (~30 kDa) protein in testis lysates from Sly-KD transgenic mice with *Sly* deficiency (sh344 and sh367). The positive control was a negative sibling of Sly-KD mice (neg sib) while the negative control was a male lacking the NPYq (NPYq-2). (**B**): Levels of protein expression shown in panel A quantified with *ImageJ* software (https://imagej.nih.gov/ij/) and normalized with respect to ACTB signal and with neg sib data serving as the normal expression baseline. (**C**,**D**) SLY expression in males with NPYq- and *Sly*-specific deficiency. (**C**) Exemplary Western blot detection of SLY1 and SLY2 protein in testis lysates from wild-type control (XY), mutant mice with progressively increasing NPYq deficiency (2/3NPYq-, 9/10NPYq-, and NPYq-2) and sh344 and sh367. (**D**) Levels of protein expression quantified with *ImageJ* software and normalized with respect to the ACTB signal and with XY data serving as the normal expression baseline. The data represent average ± SEM of several western runs with the following male number per genotype: *n* = 8, 6, 6, 6, 4, 7 for XY, 2/3NPYq-, sh344, sh367, 9/10NPYq-, and NPYq-2, respectively. Statistical significance (*t*-test): for each protein isoform, the genotypes marked with different letters are different from each other.

To further verify that the antigen is the putative SLY1 and SLY2 protein, Western blot analyses were carried out on testis extracts from males with previously reported [11,12] deficiencies in *Sly* transcript expression (Figure 1). Based on prior quantification of the *Sly* transcript level, *Sly*-deficient transgenic line sh344 was expected to have *Sly1*-specific deficiency while transgenic line sh367 was expected to be deficient for both *Sly1* and *Sly2*, and be more strongly affected overall [12]. The Western analyses were in agreement with the transcript expression data; sh344 males showed severe deficiency of SLY1 but not SLY2 while sh367 males had no SLY1 and severe loss SLY2 (Figure 1A,B).

Combined results testing the specificity of a newly developed antibody supported that it specifically recognizes SLY1 and SLY2 and is therefore appropriate to comprehensively evaluate SLY expression.

### 3.2. *SLY* Protein Expression in Mice with NPYq and Sly Deficiencies Matches Previously Reported Transcript Expression Data

The expression of SLY1 and SLY2 in the testes from males with NPYq- and *Sly*-deficiencies was examined using several males per genotype and repeated western runs (Figure 1C,D and Figure S4). Both SLY1 and SLY2 were reduced about 2-fold in the testes from males carrying a partial NPYq deletion (2/3NPYq-). Males with the deletion removing 9/10 of the NPYq (9/10NPYq-) had negligible SLY1 and SLY2 and males lacking the entire NPYq (NPYq-2) lacked both SLY isoforms. The repeated analyses of sh344 and sh367 transgenic males confirmed that the sh344 males overexpressed SLY2 and retained some of SLY1 while the sh367 males had an almost complete loss of SLY1 and very significant reduction of SLY2. Overall, the protein expression data were in agreement with the previously reported [12] decrease in transcript levels in NPYq- and *Sly*-deficient mice.

### 3.3. Addition of Flag-Sly Transgenes Rescues Sly/*SLY* Expression Deficiency in *2/3NPYq*- and *9/10NPYq*-Mice

To further investigate the role of SLY in the spermiogenic phenotype of mice with NPYq/*Sly* deficiencies, we pursued a transgene rescue strategy. First, mice transgenic for two *Sly* transcript variants, *Sly1* and *Sly2*, fused with *Flag* tag sequence and under the control of the spermatid-specific promoter *SP10*, were produced by pronuclear injection. *Sly1*- and *Sly2*-specific constructs were injected either separately (to yield single gene, *Sly1* or *Sly2*, transgenics) or together (to yield double-gene, *Sly1/2*, transgenics). The offspring derived from pronuclear injection were genotyped and two *Sly1/2* double transgenic, six *Sly1* transgenic, and two *Sly2* transgenic founders with germline transmission were obtained (Figure S5A). Male F1 and F2 progeny derived from these founders provided testes for *Sly* expression analyses. These analyses identified one *Sly1/2* (6P) and two *Sly1* (30A and 16D) transgenic lines with *Sly* overexpression 2–8-fold higher when compared to endogenous *Sly* levels (Figure S5B). These three lines were propagated to produce transgenic females.

Transgenic females were used for breeding with subfertile 2/3NPYq- males and provided oocytes for ICSI with sperm from infertile 9/10NPYq- males. The resulting NPYq-deficient male progeny were genotyped and the males with the supplementing *Flag*-*Sly* transgene/s (tsgic) and their transgene negative siblings (neg sib) were examined for the rescue of *Sly* expression.

**Figure 2:**
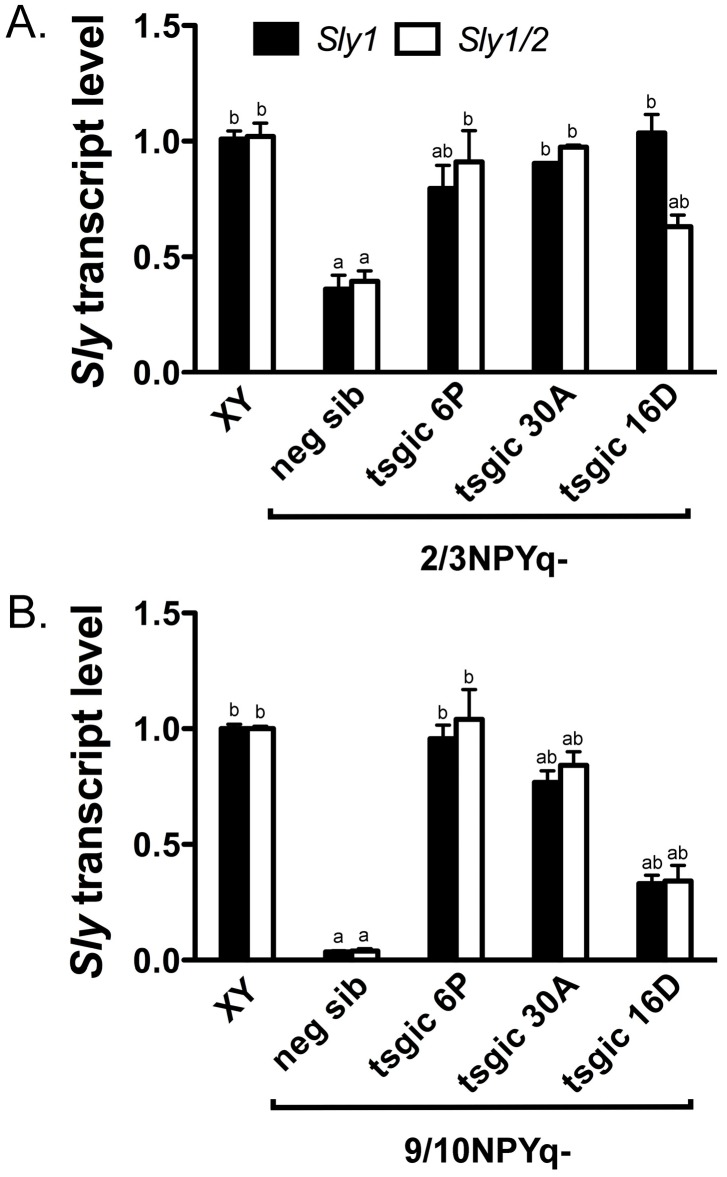
**Addition of the *Flag-Sly* transgene to males with NPYq deletions rescues *Sly* expression deficiency**. *Sly* transcripts levels (*Sly1* and *Sly1/2* global) in whole testes from moderately (**A**) and severely (**B**) NPYq-deficient mice with (tsgic) and without (neg sib) *Flag-Sly* transgene addition obtained by real-time RT-PCR with *Actb* as a loading control and normalized to wild-type XY controls. Three transgenic lines were tested: 6P carrying *Sly1* and *Sly2* transgenes and lines 30A and 16D positive for *Sly1* transgene only. There were no differences between XY and neg sib from different transgenic lines so the XY and neg sib data were pooled (the data showing all transgenic lines assayed separately is shown in Figure S6). The graphs are mean ± SEM with *n* = 14, 14, 9, 3, 4 (**A**) and *n* = 9, 9, 3, 3, 3 (**B**) for XY, neg sib, and tsgic 6P, 30A and 16D, respectively. Statistical significance (*t*-test, *p* < 0.05): ^a^ different than respective transcript type in XY; ^b^ different than respective transcript type in neg sib. Primer sequences are shown in Table S2.

The addition of the *Flag*-*Sly* transgene partially or completely rescued *Sly* expression in all three transgenic lines. When the *Flag*-*Sly* transgenes were added to 2/3NPYq-deficient mice (Figure 2A and Figure S6), all three transgenic lines had *Sly* levels significantly higher than their transgene negative siblings. When 2/3NPYq- *Flag*-*Sly* males were compared to wild-type control (XY), there were no differences in *Sly1/2* and *Sly1* levels between XY and transgenic males from line 30A (complete expression rescue) while males from transgenic lines 6P and 16D displayed partial *Sly* rescue (6P: partial *Sly1* and complete *Sly1/2*; 16D: complete *Sly1* and partial *Sly1/2*). When the *Flag*-*Sly* transgenes were added to 9/10NPYq-deficient mice (Figure 2B and Figure S6), all transgenic males had *Sly* levels significantly higher than their negative siblings. When 9/10NPYq- *Flag*-*Sly* males were compared to the wild-type control (XY), there were no differences in *Sly1/2* and *Sly1* levels between XY and transgenic males from line 6P (complete expression rescue) while transgenic males from lines 30A and 16D had partial rescue of both *Sly1/2* and *Sly1* expression.

**Figure 3:**
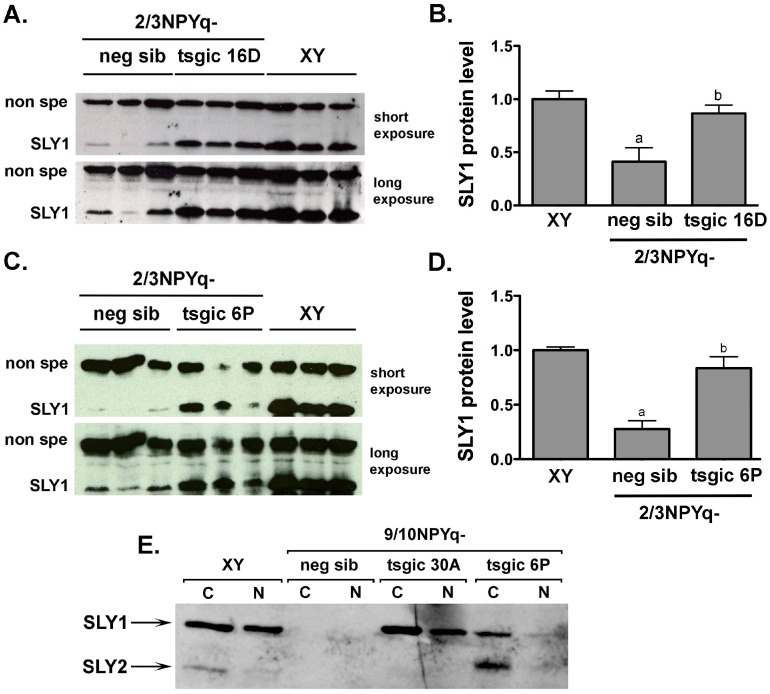
**Addition of the *Flag-Sly* transgenes to males with NPYq deficiency rescues SLY1 and SLY2 expression deficiency**. (**A**–**D**) Western blot was performed with whole testes lysates obtained from XY males and from males with moderate NPYq deficiency (2/3NPYq-) with (tsgic) and without (neg sib) *Flag-Sly* transgene addition. Two transgenic lines were tested: 16D positive for the *Sly1* transgene (**A**,**B**) and 6P positive for both the *Sly1* and *Sly2* transgenes (**C**,**D**). Levels of protein expression shown in panels (**A**,**C**) were quantified with *ImageJ* software, normalized to a non-specific band (**B**,**D**) with XY data serving as the normal expression baseline. The normalization for line 6P was also done to Ponceau signal (Figure S7). The data represent an average ± SEM with *n* = 3. Statistical significance (*t*-test, *p* < 0.05): ^a^ different from XY; ^b^ different from neg sib. (**E**) Western blot was performed with cytoplasmic (C) and nuclear (N) protein lysates from the whole testes obtained from XY males and males with severe NPYq deficiency (9/10NPYq-) with (tsgic) and without (neg sib) *Sly* transgene addition. Two transgenic lines were tested: 30A positive for the *Flag-Sly1* transgene and 6P carrying both the *Sly1* and *Sly2* transgenes. Due to the scarcity of testicular material, the reference gene was not included in this analysis.

Western blot analyses were performed to confirm that transcript expression rescue translated to the rescue of SLY protein expression. Because the production and analyses of mice with NPYq deficiency and transgene rescue preceded our development of a new anti-SLY1/2 antibody, we first tested SLY1 rescue using an anti-SLY1 antibody developed and reported on before [17]. Western blots were obtained with testis lysates from XY males and 2/3NPYq- males with (tsgic) and without (neg sib) *Flag*-*Sly* transgene addition (Figure 3 and Figure S7). Two transgenic lines were tested, 16D positive for the *Sly1* transgene and 6P positive for both the *Sly1* and *Sly2* transgenes. 2/3NPYq- males had SLY1 levels 3–4-fold lower than XY, as expected. In 2/3NPYq-*Sly* transgenic males SLY1 expression deficiency was rescued yielding SLY1 levels similar to those of XY controls (Figure 3 and Figure S7).

The rescue of SLY expression was also examined in mice with severe NPYq deficiency (9/10NPYq-) with (tsgic) and without (neg sib) *Flag*-*Sly* transgene addition, with a distinction between cytoplasmic and nuclear SLY expression to assess the transgenic SLY ability to mimic the behavior of endogenous SLY (Figure 3E). When Western blot was performed with cytoplasmic and nuclear protein lysates from whole testes obtained from wild-type XY males, SLY1 was abundantly present in both cytoplasmic and nuclear fractions, while SLY2 was much less abundant overall (Figure 3E). No SLY1 and SLY2 were detected in mice with severe NPYq deficiency (9/10NPYq-), as expected. However, when the *Flag*-*Sly* transgenes were added, the SLY1 expression was rescued in 9/10NPYq- males transgenic for *Sly1* (30A), and both SLY1 and SLY2 expression was rescued in 9/10NPYq- males transgenic for *Sly1*/*2* (6P). Similarly, as in XY, SLY2 predominated in the cytoplasmic fraction. Because of the scarcity of testicular material, only one Western blot was performed and a reference gene was not included in this analysis. However, even with this limitation, the obtained results clearly show that transgenic SLY1 and SLY2 proteins were present and capable of entering into the cell nuclei (Figure 3E).

### 3.4. Addition of Flag-Sly Transgenes Does Not Rescue Spermiogenic Phenotype of *2/3NPYq*- and *9/10NPYq*-Mice

**Figure 4:**
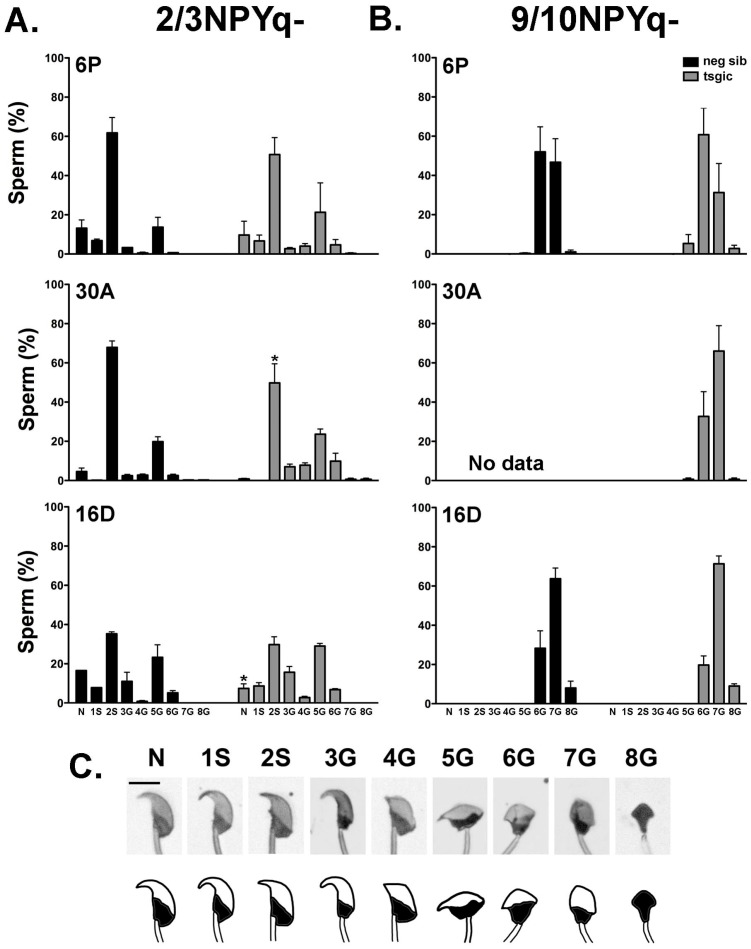
**Addition of the *Flag-Sly* transgene does not rescue sperm morphology defects in males with NPYq deficiency.** Sperm headshape was evaluated in mice with moderate (**A**, 2/3NPYq-) and severe (**B**, 9/10NPYq-) deficiency with (tsgic) and without (neg sib) the *Flag-Sly* transgene. Three transgenic lines were tested: 6P positive for both the *Sly1* and *Sly2* transgenes (top), and 30A (middle) and 16D (bottom) positive for the *Sly1* transgene. Normal headshape (N) and eight categories of headshape defects (slight: 1S-2S and gross: G3–G8) were differentiated (**C**). The data represent an average ± SDev with *n* = 2–4 males per genotype and 300 sperm examined per male (**A**) or average ± SDev with *n* = 3–4 males per genotype and 100 sperm examined per male (**B**). The photo/diagram composite (**C**) was published by us before (Figure 4 in Reference [8]); Bar = 5 µm. No data for neg sib are shown for line 30A because no transgene negative siblings were obtained in ICSI trials. Statistical significance: two-way ANOVA with genotype and sperm headshape as factors, revealed no effect of genotype (*p* > 0.05) and a strong effect of headshape (*p* < 0.0001) for all groups tested. The interaction effect (*p* < 0.05) was present only for 2/3NPYq- 30A and 16D groups. The results of paired comparison for specific sperm headshape types between transgenic and negative siblings in a post-hoc Bonferroni test are shown within the graphs: * *p* < 0.05.

2/3NPYq-*Sly* and 9/10NPYq-*Sly* males were subjected to the analyses of rescue of sub- and infertile phenotype via the analysis of the sperm parameters (number, motility and morphology) and sperm ability to fertilize oocytes in vitro. The complete characterization was done for 9/10NPYq-*Sly* males (Table 1, Figure 4) while for 2/3NPYq-*Sly* males, only sperm morphology, the most prominent spermiogenic phenotypic feature of these mice, was assessed (Figure 4).

There were no differences in body weight, testis weight, and sperm number between 9/10NPYq-*Sly* males and their transgene negative 9/10NPYq- siblings in all three transgenic lines tested (6P, 16D, and 30A). Both 9/10NPYq- and 9/10NPYq-*Sly* males had low sperm number (range: 0.1 × 10^6^–4.2 × 10^6^) typical of the 9/10NPYq- genotype, and these sperm were unable to fertilize oocytes in vitro (Table 1).

Sperm headshape defects are the most prominent feature of mice with NPYq deficiencies. The incidence of various headshape defects in mice with moderate (2/3NPYq-) and severe (9/10NPYq-) NPY deficiency with (tsgic) and without (neg sib) *Flag*-*Sly* transgenes was quantified according to criteria established by us before [8] (Figure 4). In agreement with previously published data [8,9], 2/3NPYq- mice were moderately affected with ~20% of sperm having a normal headshape and the remaining ~80% having various headshape defects, the majority of which were categorized as slight (Figure 4A), while 9/10NPYq- males had no morphologically normal sperm and all observed headshape defects were gross (Figure 4B). The presence of the *Flag*-*Sly* transgenes did not rescue sperm headshape abnormalities, with transgenic males presenting with a similar frequency and distribution of defects as their transgene negative siblings (Figure 4).

### 3.5. Addition of Flag-Sly Transgenes Does Not Rescue Gene Upregulation Associated with *2/3NPYq*- and *9/10NPYq*-Deficiency

**Figure 5:**
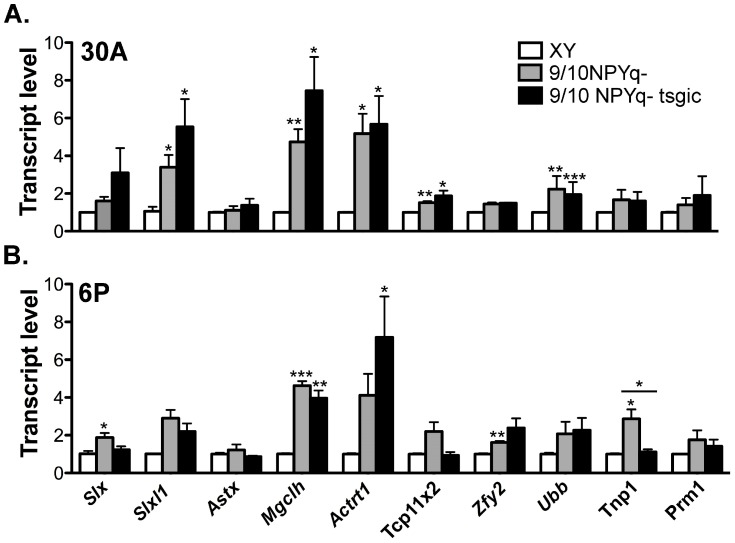
**Addition of the *Flag-Sly* transgene to males with NPYq deletions does not rescueX-Y gene upregulation**. Gene expression in testes from NPYq-deficient mice without (9/10NPYq-) and with (9/10NPYq- tsgic) the *Flag-Sly* transgene was obtained by real-time RT-PCR with *Actb* as a loading control and normalized to wild-type XY controls. Two transgenic lines were analyzed: line 30A carrying the *Sly1* transgene (**A**) and line 6P carrying both the *Sly1* and *Sly2* transgene (**B**). NPYq- deficient mice without the transgenes were negative siblings of transgenics. The graphs are mean ± SEM with *n* = 3. Statistical significance (*t*-test): * < 0.05; ** < 0.01; *** < 0.001, in comparison with NPYq- deficient mice with XY; the difference between transgenics and their negative siblings is marked with a horizontal lane above the relevant graph pair. Primer sequences are shown in Table S2.

NPYq- and *Sly*-deficient males were shown before to display a remarkable upregulation of sex chromosome genes after meiosis, and we proposed that the spermiogenic defects associated with NPYq/*Sly* deficiency might be a consequence of the massive and global upregulation of spermiogenic genes [11,12]. Since 2/3NPYq-*Sly* and 9/10NPYq-*Sly* males displayed a similar spermiogenic phenotype as their non-transgenic siblings, we predicted that both types of males will also present with the X and Y gene upregulation. The analysis of expression of several X (*Slx*, Sycp3 like X-linked; *Slxl1*, Slx-like 1; *Astx*, Amplified spermatogenic transcript X encoded 5; *Mgclh*, Germ cell-less protein-like 2; *Actrt1*, Actin-related protein T1; *Tcp11x2*, T-complex 11 family, X-linked 2), Y (*Zfy2*, Zinc finger protein 2, Y-linked) and autosomally (*Ubb*, Ubiquitin B; *Tnp1*, Transition protein 1; *Prm1*, Protamine 1) encoded genes revealed that genes were upregulated in both 9/10NPYq- and 9/10NPYq-*Sly* males (Figure 5).

### 3.6. Flag-Sly Transgenic Line Generated and Analyzed Independently Confirms that Addition of the Sly1 Transgene Does Not Rescue Spermiogenic Phenotype of Mice with *NPYq* Deficiency

**Figure 6:**
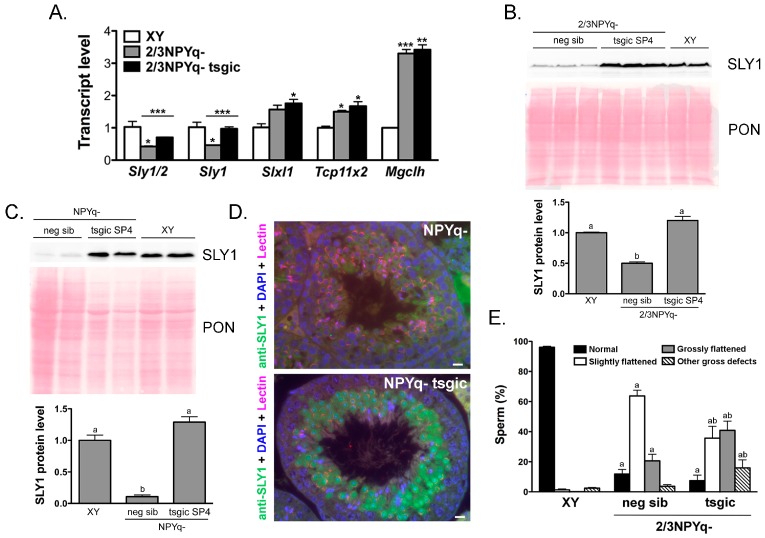
Flag-*Sly1* SP4 transgenic line generated and analyzed independently confirms that the rescue of *Sly1* expression does not lead to the rescue of the spermiogenic phenotype of mice with NPYq deficiency. (**A**) Gene expression in testes from NPYq-deficient mice without (2/3NPYq-) and with (2/3NPYq- tsgic) the *Flag*-*Sly1* SP4 transgene was obtained by real-time RT-PCR with *Acrv1* as a loading control and normalized to wild-type XY controls. The graphs are mean ± SEM with *n* = 3. Statistical significance (*t*-test): * < 0.05; *** < 0.001, in comparison with NPYq-deficient mice with XY; the difference between transgenics and their negative siblings is marked with a horizontal lane above the relevant graph pairs. Primer sequences are shown in Table S2. (**B**,**C**) SLY1 expression rescue shown with Western blot performed with whole testes lysates obtained from males with moderate NPYq deficiency (2/3NPYq-) (**B**) or from males lacking the entire Y chromosome long arm (NPYq-) (**C**) with (tsgic SP4) and without (neg sib) the addition of the *Flag*-*Sly1* SP4 transgene. Levels of protein expression were quantified with *ImageJ* software and normalized to Ponceau (PON) signal. The data represent an average ± SDev, with *n* = 2–3. Statistical significance (*t*-test, *p* < 0.05): bars marked with different letters are significantly different. D: Immunofluorescence detection of transgenic SLY1 protein (green) on stage VII testicular tubules from mice lacking the entire Y chromosome long arm without (NPYq-) and with (NPYq- tsgic) the *Flag*-*Sly1* SP4 transgene. Lectin (red) was used to stage the tubules and DAPI (blue) was used to stain the nuclei. Bar = 10 µm. (**E**) Sperm headshape analysis performed for NPYq-deficient mice without (2/3NPYq-) and with (2/3NPYq- tsgic) the *Flag*-*Sly1* SP4 transgene, and XY controls. Normal headshape and three categories of headshape defects were differentiated and quantified. The data represent an average ± SEM with *n* = 13, 8, and 3 males of the XY, 2/3NPYq-, and 2/3NPYq- tsgic genotype; a total of 1227, 651, and 274 sperm were examined for XY, 2/3NPYq-, and 2/3NPYq- tsgic genotype, respectively. Statistical significance: two-way ANOVA with genotype and sperm headshape as factors revealed a significant effect of genotype (*p* = 0.0034), headshape (*p* < 0.0001) and interaction (*p* < 0.0001). The results of paired comparison for specific sperm headshape between transgenic and negative siblings in post-hoc Bonferroni test are shown within graphs: ^a^ *p* < 0.05 when compared to XY and ^b^ *p* < 0.05 when compared to 2/3NPYq-.

All data presented thus far were obtained with transgenic and NPYq/*Sly* deficient mice produced in Hawaii (Ward Lab). Independently, in France (Cocquet Lab), another line of mice transgenic for *Flag*-*Sly1*, SP4, were generated and examined. Transgenic XY SP4 males had *Sly1* and global *Sly1/2* transcript levels elevated 2–2.5-fold compared to non-transgenic siblings and the SP4 *Sly1* transgene recapitulated all features of endogenous SLY1 in the XY context [14]. When the SP4 *Flag*-*Sly1* transgene was added to 2/3NPYq- mice, *Sly1* expression was rescued at the transcript level (Figure 6A). Western blot analyses were performed and showed that the SLY1 protein level is also rescued with a protein level similar in 2/3NPYq- with the SP4 *Sly1* transgene and in WT (XY) testes (Figure 6B). When the SP4 *Flag*-*Sly1* transgene was added to mice lacking all NPYq genes (NPYq-), the SLY1 protein level of expression was also rescued (Figure 6C) and the pattern of transgenic FLAG-SLY1 expression was similar to that of endogenous SLY1 in XY testes, i.e., strong nuclear and cytoplasmic signal in steps 2/3 to 9 spermatids (Figure 6D) [11,17]. The presence of the SP4 *Flag*-*Sly1* transgene in 2/3NPYq- males did not rescue the sperm headshape defects (Figure 6E) and derepression of X encoded genes *Slxl1*, *Tcp11x2* and *Mgclh* (Figure 6A). Similarly, SP4 *Flag*-*Sly1* transgene in NPYq- males did not rescue testis weight nor sperm number (average NPYq- SP4 testis weight = 67.5 mg vs. WT = 102.3 mg; average NPYq- SP4 sperm number per cauda epididymis = 0.45 × 10^6^ vs. WT = 13.6 × 10^6^). Altogether, these data confirm by an independent study that the rescue of SLY1 expression level and pattern with a *Flag-Sly1* transgene does not rescue the NPYq deficiency phenotype.

## 4. Discussion

The goal of this study was to clarify whether the spermiogenic phenotype of mice with NPYq deficiencies was due to the absence of NPYq-encoded *Sly* gene. Prior analyses of mice with transgenically silenced *Sly* [11,12] provided strong evidence to support this hypothesis. However, one unresolved issue was whether *Sly* deficiency was the sole underlying cause. Here, we provide new data indicating that the spermiogenic phenotype in NPYq/*Sly* deficient mice correlates well with the deficiency of expression of endogenous *Sly*/SLY. However, transgenic rescue of *Sly/*SLY expression impairment in NPYq-deficient mice does not ameliorate their spermiogenic phenotype, suggesting that another NPYq encoded gene contributes to the spermiogenic phenotype.

### 4.1. New anti-*SLY* Antibody Confirms that Some *SLY* Protein is Retained in sh367 Sly-KD Mice 

*Sly* (Sycp3-like Y-linked) is one of the genes encoded within a massively amplified region of the mouse Y chromosome; 126 copies of *Sly* with intact open reading frames are present on NPYq [1]. *Sly* encodes two main transcript variants, *Sly1* and *Sly2* [17]. *Sly1* is a full-length isoform and encodes a ~40-kDa protein SLY1 while *Sly2* originates from the alternative splicing of exons 5 and 6 and is translated to produce the shorter protein SLY2. Interestingly, exons 5 and 6 are duplicates of exons 3 and 4, and the functional difference (if any) between SLY1 and SLY2 remains unknown. Not all *Sly* copies on NPYq have the same structure and the picture is further complicated by additional copies expected to produce shorter ORF and some being non-coding.

The characterization of SLY protein(s) has to date focused on its most predominant isoform: SLY1. SLY1 has been shown to be very highly expressed in round spermatids, steps 2/3 to 9, with nuclear localization and, in particular, co-localization with sex chromosomes and *Speer* autosomal cluster during spermiogenesis [11,30]. The SLY protein contains a conserved COR1 domain initially identified in the synaptonemal complex protein SYCP3 and is able to interact with double-stranded DNA [31,32]. By chromatin immunoprecipitation, SLY1 protein has been shown to be enriched at the promoter of many spermatid-expressed genes, including spermatid-specific multicopy X and Y and genes involved in chromatin remodeling during spermiogenesis [14].

The sh367 Sly-KD mice were shown previously to have the most prominent *Sly* knockdown, sperm with gross head abnormalities, and severely impaired fertility [11]. These mice also have an increased incidence of DNA breaks in sperm and impaired sperm chromatin packaging [12]. The spermiogenic phenotype of sh367 mice was less pronounced than that of mice with severe NPYq deficiencies [11,12]. One of the intermediate *Sly* knockdown lines, line sh344, was shown to have moderate *Sly* transcript knockdown, no sperm headshape defects, good fertility, and no sperm DNA damage phenotype [12] While decreasing *Sly* transcript levels correlated well with the increasing severity of the phenotype, protein expression analyses were hampered by a lack of an appropriate antibody [30,31,32]. Here, using a new anti-SLY antibody allowing for the distinction between SLY1 and SLY2, we demonstrated that sh367 mice retain some SLY2, which could be responsible for their mild phenotype. We also demonstrated that phenotypically unaffected sh344 males lack most SLY1 but retain (and overexpress) SLY2, which emphasizes the role of the SLY2 isoform.

The fact that *Sly2* transcripts and protein are overexpressed when *Sly1* transcripts are knocked down is in agreement with observations that SLY negatively regulates its own expression [11,14]. Because the SLY2 sequence does not have anything unique compared to that of SLY1 and the alternatively spliced out region is in fact duplicated in SLY1, it is tempting to say that SLY1 and SLY2 have a similar function. The fact that *Sly* and NPYq- deficient phenotypes correlate well with *Sly1/2* transcript and SLY1/2 protein levels is further evidence in favor of this hypothesis (Table S3). Yet, only the production of an anti-SLY2 specific antibody, if feasible, will allow for confirmation of this assumption.

### 4.2. Transgenic *SLY* Rescues Sly/*SLY* Expression But Not the *NPYq* Specific Spermiogenic Phenotype, Suggesting the Involvement of Another *NPYq* Gene in the Same Pathway 

Transgene rescue, along with gene knockout/knockdown, is a viable and commonly used approach for establishing gene function [33,34,35,36]. Therefore, to further elucidate whether the loss of *Sly* is solely responsible for the spermiogenic phenotype of mice with NPY deficiencies, we generated males transgenic for *Sly* and examined the effects of placing the transgene in the context of varying degrees of NPYq deficiencies ranging from 2/3NPYq to complete deletion of the NPYq region. In all the lines we produced, despite reaching a global level of *Sly1/2* transcripts and SLY1/2 proteins at least equal (and often superior) to what is observed in WT controls, we did not see any amelioration in the parameters we looked at: sperm morphology, number, motility, fertilizing ability, as well as post-meiotic sex chromatin (PMSC) gene expression.

What could be the reasons for the inability of transgenic SLY to overcome the spermiogenic phenotype of mice with NPYq deficiencies? One could suspect FLAG tag to interfere with the SLY protein function. However, immunoprecipitation and chromatin immunoprecipitation analyses performed with XY mice carrying the FLAG tagged transgenic SLY1 did not show any difference between FLAG-SLY1 transgenic protein and SLY1 endogenous protein in regard to binding partners and targets [14]. To further challenge this hypothesis, we generated mice transgenic for *Sly* but without the FLAG tag. The transgenic rescue with the *Sly* transgenes lacking the tag yielded similar results to the transgenes with *Flag-Sly* (Figure S8).

Another potential reason for the lack of spermiogenic phenotype rescue could be the timing of transgenic *Sly*/SLY expression. We selected the *SP10* promoter to drive the *Sly* transgenes in order to achieve a high spermatid-specific expression level from step 1 round spermatids [37,38]. As shown here and before [14], the *SP10* promoter drives the expression of transgenic *Sly* with a pattern very similar to that of endogenous *Sly*. Though slight differences may exist, it would more likely decrease the level of phenotypic rescue, rather than lead to a complete lack of it.

One can also ask whether the lack of phenotype rescue in lines carrying the *Flag-Sly1* transgene is due to a lack of expression rescue of *Sly2*/SLY2. Two pieces of evidence go against it. First, the results obtained with line 6P carrying both the *Flag-Sly1* and *Flag-Sly2* transgenes and showing both *Sly1* and *Sly2* transcripts and SLY1 and SLY2 protein expression rescue, presented no phenotype rescue. Second, the experiments performed with transgenic rescue mice carrying the *Sly1* and *Sly2* transgenes without *Flag*, showed that the addition of *Sly2* rescued the expression but did not rescue the phenotype (Figure S8).

All in all, we believe that the most likely reason for the lack of NPYq rescue with the *Sly* transgenes is that another NPYq encoded gene/s are involved in the same pathway. The most likely candidate is the multi-copy gene *Ssty,* which has been shown to be reduced in mice with NPY deficiencies [2]. On NPYq, 306 copies of *Ssty* with intact open reading frame have been identified [1]. Two versions of the *Ssty* gene (i.e., *Ssty1* and *Ssty2*) have been described and, as for *Sly*, a related spermatid-specific multicopy gene family exists on the X chromosome [39,40]. SSTY proteins belong to the SPIN family, all members of which bear three Spin/Ssty domains. One member of this family, SPIN1, has been extensively studied and was found to homo-dimerize and be able to recognize H3K4me3 and H3R8me2a [40,41].

SSTY molecular function has not been fully investigated, but we have shown that SSTY proteins are specifically expressed in spermatids, co-localize with PMSC, and interact with SLY and its X chromosome-linked homologues SLX/SLXL1 [42]. In a study in which H3K4me3 peptide was pulled down to identify chromatin readers and associated proteins, SSTY1, SSTY2, SLX/SLXL1, and SLY were found as part of the protein complexes interacting with H3K4me3 specifically in the testis [43], suggesting that SSTY proteins, like SPIN1, could bind to H3K4me3. As discussed in our previous studies [11,29], the SLY isoelectric point is acidic and, therefore, is not compatible for a direct interaction with DNA. Yet, ChIP-Seq analyses revealed that SLY1 genomic location overlaps with that of H3K4me3 in round spermatids [14]. A very tempting hypothesis is that SSTY proteins (possibly in the form of dimers) are required for SLY to interact with DNA/chromatin and control gene expression. According to this model, in a context in which both *Sly* and *Ssty* levels are decreased or absent, rescuing the sole expression of *Sly* would not improve the NPYq phenotype due to a lack of (enough) *Ssty*. Interestingly, the NPYq structure consists mostly of the repetition (in tandem or palindromic) of a ~500 kb unit containing one copy of *Sly* and three of *Ssty1/2* [1,44]. In the mouse lineage, they have been co-amplified [45], thus maintaining a similar stoichiometry between *Sly* and *Ssty* gene copies/amount of proteins.

Future work will involve testing for the *Ssty* roles by generating mice with *Ssty* knockdown as we have done with *Sly*. Transgene rescue of NPYq deficiency with *Ssty* transgenes should also be informative. We anticipate that combining the *Sly* and *Ssty* transgenic rescue might provide the most efficient reconstitution of normal spermatogenesis and fertility in a context of severe NPYq deficiency.

## 5. Conclusions

In conclusion, the transgenic rescue we used in this study is an interesting complementary approach to the gene knockout/knockdown strategy used before to investigate the NPYq gene function. Despite the previously established involvement of *Sly* in the NPYq- spermiogenic phenotype, a lack of rescue with the *Sly* transgene strongly suggests the participation of another NPYq gene. The best candidate identified to date is *Ssty*, which we suspect is required together with *Sly* for normal gene expression and chromatin regulation during spermiogenesis.

## Figures and Tables

**Table 1:** Addition of the *Flag-Sly* transgene to males with severe NPYq deficiency does not rescue low sperm number and sperm ability to fertilize oocytes in vitro.

Tg Line	Genotype	No Males	Age (Weeks)	Average ± SEM			Eggs Fertilized/Eggs Inseminated (%)
				Body Weight (g)	Testis Weight (mg)	Sperm Number (1CE, ×10^6^)	
6P	9/10NPYq-*Sly1/2*	4	10	27 ± 1	58 ± 4	0.1 ± 0.1	0/236 (0)
	9/10NPYq-	3	10	26 ± 2	60 ± 3	1.9 ± 0.9	0/167 (0)
	WT IVF control	2	16	34 ± 0	92 ± 3	n/a	75/121 (62)
16D	9/10NPYq-*Sly1*	4	12	32 ± 4	79 ± 10	2.5 ± 1.2	1/236 (0)
	9/10NPYq-	3	12	27 ± 1	81 ± 2	4.2 ± 2.4	0/166 (0)
	WT IVF control	2	14	29 ± 1	94 ± 3	n/a	114/122 (93)
30A	9/10NPYq-*Sly1*	3	10	26 ± 1	64 ± 4	0.4 ± 0.03	0/129 (0)
	WT IVF control	1	16	38	99, 100	n/a	39/49 (80)

Tg = transgene. 1CE = 1 cauda epididymis. There were no statistically significant differences between 9/10NPYq-*Sly* transgenics and their negative siblings 9/10NPYq- for any of the factors tested.

## Supplementary Materials

The following are available online at https://www.mdpi.com/2073-4425/10/2/133/s1, Figure S1: Design of anti-SLY antibody, Figure S2: Characterization of anti-SLY antibody, Figure S3: Dot-blot analysis, Figure S4: SLY expression in males with NPYq- and Sly-specific deficiency, Figure S5: Production of mice transgenic for *Flag-Sly*, Figure S6: Addition of the *Flag-Sly* transgene to males with NPYq deletions rescues *Sly* expression deficiency, Figure S7: Addition of the *Flag-Sly* transgene to 2/3NPYq-males rescues SLY1 expression deficiency, Figure S8: Addition of the *Sly* transgene (no FLAG tag) to males with severe NPYq deficiency rescues *Sly* expression but not low sperm number and sperm ability to fertilize oocytes in vitro, Table S1: Summary of mice used in this study, Table S2: Primers, Table S3: Relationship between SLY1/2 protein expression and spermiogenic phenotype and fertility of mice with NPY/Sly deficiency.

## References

[1] Soh Y.Q., Alfoldi J., Pyntikova T., Brown L.G., Graves T., Minx P.J., Fulton R.S., Kremitzki C., Koutseva N., Mueller J.L. (2014). Sequencing the mouse y chromosome reveals convergent gene acquisition and amplification on both sex chromosomes. Cell.

[2] Toure A., Clemente E.J., Ellis P., Mahadevaiah S.K., Ojarikre O.A., Ball P.A., Reynard L., Loveland K.L., Burgoyne P.S., Affara N.A. (2005). Identification of novel Y chromosome encoded transcripts by testis transcriptome analysis of mice with deletions of the Y chromosome long arm. Genome Biol..

[3] Burgoyne P.S., Mahadevaiah S.K., Sutcliffe M.J., Palmer S.J. (1992). Fertility in mice requires X-Y pairing and a Y-chromosomal “spermiogenesis” gene mapping to the long arm. Cell.

[4] Moriwaki K., Suh D.S. (1988). Genetic factors affecting sperm morphology in the mouse. Mouse Newsl..

[5] Styrna J., Imai H.T., Moriwaki K. (1991). An increased level of sperm abnormalities in mice with a partial deletion of the Y chromosome. Genet. Res..

[6] Styrna J., Klag J., Moriwaki K. (1991). Influence of partial deletion of the Y chromosome on mouse sperm phenotype. J. Reprod. Fertil..

[7] Toure A., Szot M., Mahadevaiah S.K., Rattigan A., Ojarikre O.A., Burgoyne P.S. (2004). A new deletion of the mouse Y chromosome long arm associated with the loss of Ssty expression, abnormal sperm development and sterility. Genetics.

[8] Yamauchi Y., Riel J.M., Wong S.J., Ojarikre O.A., Burgoyne P.S., Ward M.A. (2009). Live offspring from mice lacking the Y chromosome long arm gene complement. Biol. Reprod..

[9] Ward M.A., Burgoyne P.S. (2006). The effects of deletions of the mouse Y chromosome long arm on sperm function—Intracytoplasmic sperm injection (ICSI)-based analysis. Biol. Reprod..

[10] Yamauchi Y., Riel J.M., Stoytcheva Z., Burgoyne P.S., Ward M.A. (2010). Deficiency in mouse Y chromosome long arm gene complement is associated with sperm DNA damage. Genome Biol..

[11] Cocquet J., Ellis P.J., Yamauchi Y., Mahadevaiah S.K., Affara N.A., Ward M.A., Burgoyne P.S. (2009). The multicopy gene Sly represses the sex chromosomes in the male mouse germline after meiosis. PLoS Biol..

[12] Riel J.M., Yamauchi Y., Sugawara A., Li H.Y., Ruthig V., Stoytcheva Z., Ellis P.J., Cocquet J., Ward M.A. (2013). Deficiency of the multi-copy mouse Y gene Sly causes sperm DNA damage and abnormal chromatin packaging. J. Cell Sci..

[13] Ellis P.J., Clemente E.J., Ball P., Toure A., Ferguson L., Turner J.M., Loveland K.L., Affara N.A., Burgoyne P.S. (2005). Deletions on mouse Yq lead to upregulation of multiple X- and Y-linked transcripts in spermatids. Hum. Mol. Genet..

[14] Moretti C., Serrentino M.E., Ialy-Radio C., Delessard M., Soboleva T.A., Tores F., Leduc M., Nitschke P., Drevet J.R., Tremethick D.J. (2017). SLY regulates genes involved in chromatin remodeling and interacts with TBL1XR1 during sperm differentiation. Cell Death Differ..

[15] Reynard L.N., Turner J.M. (2009). Increased sex chromosome expression and epigenetic abnormalities in spermatids from male mice with Y chromosome deletions. J. Cell Sci..

[16] Lavery R., Chassot A.A., Pauper E., Gregoire E.P., Klopfenstein M., de Rooij D.G., Mark M., Schedl A., Ghyselinck N.B., Chaboissier M.C. (2012). Testicular differentiation occurs in absence of R-spondin1 and Sox9 in mouse sex reversals. PLoS Genet..

[17] Reynard L.N., Cocquet J., Burgoyne P.S. (2009). The multi-copy mouse gene Sycp3-like Y-linked (Sly) encodes an abundant spermatid protein that interacts with a histone acetyltransferase and an acrosomal protein. Biol. Reprod..

[18] Akerfelt M., Henriksson E., Laiho A., Vihervaara A., Rautoma K., Kotaja N., Sistonen L. (2008). Promoter ChIP-chip analysis in mouse testis reveals Y chromosome occupancy by HSF2. Proc. Natl. Acad. Sci. USA.

[19] Garcia M.A., Collado M., Munoz-Fontela C., Matheu A., Marcos-Villar L., Arroyo J., Esteban M., Serrano M., Rivas C. (2006). Antiviral action of the tumor suppressor ARF. EMBO J..

[20] Kimura Y., Yanagimachi R. (1995). Intracytoplasmic sperm injection in the mouse. Biol. Reprod..

[21] Chatot C.L., Ziomek C.A., Bavister B.D., Lewis J.L., Torres I. (1989). An improved culture medium supports development of random-bred 1-cell mouse embryos in vitro. J. Reprod. Fertil..

[22] Quinn P., Barros C., Whittingham D.G. (1982). Preservation of hamster oocytes to assay the fertilizing capacity of human spermatozoa. J. Reprod. Fertil..

[23] Ajduk A., Yamauchi Y., Ward M.A. (2006). Sperm chromatin remodeling after intracytoplasmic sperm injection differs from that of in vitro fertilization. Biol. Reprod..

[24] Ward M.A., Yanagimachi R. (2018). Intracytoplasmic Sperm Injection in Mice. Cold Spring Harb. Protoc..

[25] Burgoyne P.S., Mahadevaiah S.K., Perry J., Palmer S.J., Ashworth A. (1998). The Y* rearrangement in mice: New insights into a perplexing PAR. Cytogenet. Cell Genet..

[26] Cattanach B.M. (1987). Sex-reversed mice and sex determination. Ann. N. Y. Acad. Sci..

[27] Cattanach B.M., Pollard C.E., Hawker S.G. (1971). Sex-reversed mice: XX and XO males. Cytogenetics.

[28] Eicher E.M., Hale D.W., Hunt P.A., Lee B.K., Tucker P.K., King T.R., Eppig J.T., Washburn L.L. (1991). The mouse Y* chromosome involves a complex rearrangement, including interstitial positioning of the pseudoautosomal region. Cytogenet. Cell Genet..

[29] Comptour A., Moretti C., Serrentino M.E., Auer J., Ialy-Radio C., Ward M.A., Toure A., Vaiman D., Cocquet J. (2014). SSTY proteins co-localize with the post-meiotic sex chromatin and interact with regulators of its expression. FEBS J..

[30] Cocquet J., Ellis P.J., Mahadevaiah S.K., Affara N.A., Vaiman D., Burgoyne P.S. (2012). A genetic basis for a postmeiotic X versus Y chromosome intragenomic conflict in the mouse. PLoS Genet..

[31] Syrjanen J.L., Pellegrini L., Davies O.R. (2014). A molecular model for the role of SYCP3 in meiotic chromosome organisation. eLife.

[32] Yuan L., Liu J.G., Zhao J., Brundell E., Daneholt B., Hoog C. (2000). The murine SCP3 gene is required for synaptonemal complex assembly, chromosome synapsis, and male fertility. Mol. Cell.

[33] Mazeyrat S., Saut N., Grigoriev V., Mahadevaiah S.K., Ojarikre O.A., Rattigan A., Bishop C., Eicher E.M., Mitchell M.J., Burgoyne P.S. (2001). A Y-encoded subunit of the translation initiation factor Eif2 is essential for mouse spermatogenesis. Nat. Genet..

[34] Vernet N., Mahadevaiah S.K., Ellis P.J., de Rooij D.G., Burgoyne P.S. (2012). Spermatid development in XO male mice with varying Y chromosome short-arm gene content: evidence for a Y gene controlling the initiation of sperm morphogenesis. Reproduction.

[35] Yamauchi Y., Riel J.M., Ruthig V.A., Ortega E.A., Mitchell M.J., Ward M.A. (2016). Two genes substitute for the mouse Y chromosome for spermatogenesis and reproduction. Science.

[36] Yamauchi Y., Riel J.M., Stoytcheva Z., Ward M.A. (2014). Two Y genes can replace the entire Y chromosome for assisted reproduction in the mouse. Science.

[37] Reddi P.P., Shore A.N., Shapiro J.A., Anderson A., Stoler M.H., Acharya K.K. (2003). Spermatid-specific promoter of the SP-10 gene functions as an insulator in somatic cells. Dev. Biol..

[38] Reddi P.P., Flickinger C.J., Herr J.C. (1999). Round spermatid-specific transcription of the mouse SP-10 gene is mediated by a 294-base pair proximal promoter. Biol. Reprod..

[39] Laval S.H., Reed V., Blair H.J., Boyd Y. (1997). The structure of DXF34, a human X-linked sequence family with homology to a transcribed mouse Y-linked repeat. Mamm. Genome.

[40] Oh B., Hwang S.Y., Solter D., Knowles B.B. (1997). Spindlin, a major maternal transcript expressed in the mouse during the transition from oocyte to embryo. Development.

[41] Su X., Zhu G., Ding X., Lee S.Y., Dou Y., Zhu B., Wu W., Li H. (2014). Molecular basis underlying histone H3 lysine-arginine methylation pattern readout by Spin/Ssty repeats of Spindlin1. Genes Dev..

[42] Toure A., Grigoriev V., Mahadevaiah S.K., Rattigan A., Ojarikre O.A., Burgoyne P.S. (2004). A protein encoded by a member of the multicopy Ssty gene family located on the long arm of the mouse Y chromosome is expressed during sperm development. Genomics.

[43] Eberl H.C., Spruijt C.G., Kelstrup C.D., Vermeulen M., Mann M. (2013). A map of general and specialized chromatin readers in mouse tissues generated by label-free interaction proteomics. Mol. Cell.

[44] Ellis P.J., Ferguson L., Clemente E.J., Affara N.A. (2007). Bidirectional transcription of a novel chimeric gene mapping to mouse chromosome Yq. BMC Evol. Biol..

[45] Ellis P.J., Bacon J., Affara N.A. (2011). Association of Sly with sex-linked gene amplification during mouse evolution: A side effect of genomic conflict in spermatids?. Hum. Mol. Genet..

