# Supplementary material for: Rescue of Sly Expression Is Not Sufficient to Rescue Spermiogenic Phenotype of Mice with Deletions of Y Chromosome Long Arm
Source: Genes (Basel). 2019 Feb 12;10(2):133. doi: 10.3390/genes10020133 (PMC6409976; doi:10.3390/genes10020133)
Supplement: Supplementary file 1 [file genes-10-00133-s001.pdf]

Rescue of *Sly* expression is not sufficient to rescue spermiogenic phenotype of mice with deletions of Y chromosome long arm

Jonathan M. Riel [1], Yasuhiro Yamauchi [1], Victor A. Ruthig [1], Qushay Malinta [1], Mélina Blanco [2], Charlotte Moretti [2], Julie Cocquet [2] and Monika A. Ward [1,*]

SUPPLEMENTARY MATERIAL

Figure S1: Design of anti-SLY antibody.

Figure S2: Characterization of anti-SLY antibody.

Figure S3: Dot-blot analysis.

Figure S4: SLY expression in males with NPYq- and *Sly*-specific deficiency.

Figure S5: Production of mice transgenic for *Flag-Sly*.

Figure S6: Addition of the *Flag-Sly* transgene to males with NPYq deletions rescues *Sly* expression deficiency.

Figure S7: Addition of the *Flag-Sly* transgene to 2/3NPYq- males rescues SLY1 expression deficiency.

Figure S8: Addition of the *Sly* transgene (no FLAG tag) to males with severe NPYq deficiency rescues *Sly* expression but not low sperm number and sperm ability to fertilize oocytes in vitro.

Table S1: Summary of mice used in this study.

Table S2: Primers.

Table S3: Relationship between SLY1/2 protein expression and spermiogenic phenotype and fertility of mice with NPY/Sly deficiency.

```
SLY1     MRRMALKKLKVIPKEGYLLLLDFDDEDDDIKVSEEALSEVKSPAFDKNENISPQAEADED  60
SLY2     MRRMALKKLKVIPKEGYLLLLDFDDEDDDIKVSEEALSEVKSPAFDKNENISPQAEADED  60
SLX      ---MSIKKLWVIPKDGYLLLLDFDSDEEEEQ----AHSEVKRPAFGKHENMPPHVEADED  53
SLXL1    ---MALKKLWAIPKDGYLLLLDYDDEDDDIN----FLE----------------------  31
XLR      --------------------------------------------------MENWDLSSDE  10
SYCP3    ------------------MLRGCGDSDSS------------PEPLSKHLKMVPGGRK---  27

SLY1     MGDE------------------------------------------VDSMLDKSEVNNPA  78
SLY2     MGDE------------------------------------------VDSM----------  68
SLX      IRDEQDSMLDKSGENVSFSVEWQRFARSVETPMENWNLLSGEQQVRNASELDLMEVQNPV  113
SLXL1    --------DAHSEENVSFSEEWQRFASSVETPIENRNLLSGEQQDGNASKLDLMEEQNPV  83
XLR      MQDG------------------------------------------NAPELDVIEEHNPV  28
SYCP3    -------HSGKSGK-----------PPLVDQPKKAFDFEK-DDKDLSGSEEDVADEKAPV  68

SLY1     IGKDENISPQVKGDEDMGHEVGSMLDKSGDDIYKTLHIKRKWMETYVKESFKGSNQKLER  138
SLY2     -------------------------LDKSEDDIYKTLHIKRKWMETYVKESFKGSNQKLER  104
SLX      THDDGNANPEVVV-----------------------------------------------  126
SLXL1    THDDENEIPEEIV-----------------------------------------------  96
XLR      TRDDENANPEEVV-GDTRSPVQNILGKFEGDINKRLHIKRKRMETYIKDSFKDSNVKLEQ  87
SYCP3    IDKHGKKRSAG-IIEDVGGEVQNMLEKFGADINKALLAKRKRIEMYTKASFKASNQKIEQ  127

SLY1     FCKTNERERKNINNKFCEQYITTFQKSDMDVQKFNEEKEKSVNSCQKEQQALKLSKCSQN  198
SLY2     FCKTNERERKNINNKFCEQYITTFQKSDMDVQKFNEEKEKSVNSCQKEQQALKLSKCSQN  164
SLX      -----GDTRKKINNKLCEQ------KFDMDIQKFNEEQEKSVNNYQKEQQALKLFECSQS  175
SLXL1    -----GDTREMINNKSCEQYKTTFQKFDMDVQNFNEQQEKS-------------------  132
XLR      LWKTNKQERKKINNKFCEQYITTFQKFDMDVQKFNEEQEKSVNNYQKEQQALKLSKCSQS  147
SYCP3    IWKTQQEEIQKLNNEYSQQFMNVLQQWELDIQKFEEQGEKLSNLFRQQQKIFQQSRIVQS  187
                  : :**: .:*      : ::*:*:*:*: **

SLY1     QTLEAVKEMHEKSMEVLMNLGTKN------------------------------------  222
SLY2     QTLEAVKEMHEKSMEVLMNLGTKN------------------------------------  188
SLX      QTLEAIEDMHEKSMEGLMNMETNNYDMLFDVDGEETL-----------------------  212
SLXL1    --------------VGLMNLETNNSDMLFDVDGELRK-----------------------  155
XLR      QTLEAIKDMHENYMEGLMNLETNNYNMLFDVDGELRKEMSVFKKDLMKHTLKYSSSFPSS  207
SYCP3    QRMFAMKQIHEQFIKSLEDVEKNNDNLFTGTQSELKKEMAMLQKKVMMETQQQEMANVRK  247
                         * :: .:*

SLY1     -------  222
SLY2     -------  188
SLX      -------  212
SLXL1    -------  155
XLR      D------  208
SYCP3    SLQSMLF  254
```

Figure S1. Design of anti-SLY antibody. A ClustalW alingment of the SLY1 and SLY2 amino acid sequences with the related proteins SLX, SLXL1, XLR, and SYCP3. The 15 amino acid specific peptide VKSPAFDKNENISPQ (red) was used to immunize mice to produce an anti-SLY antibody.

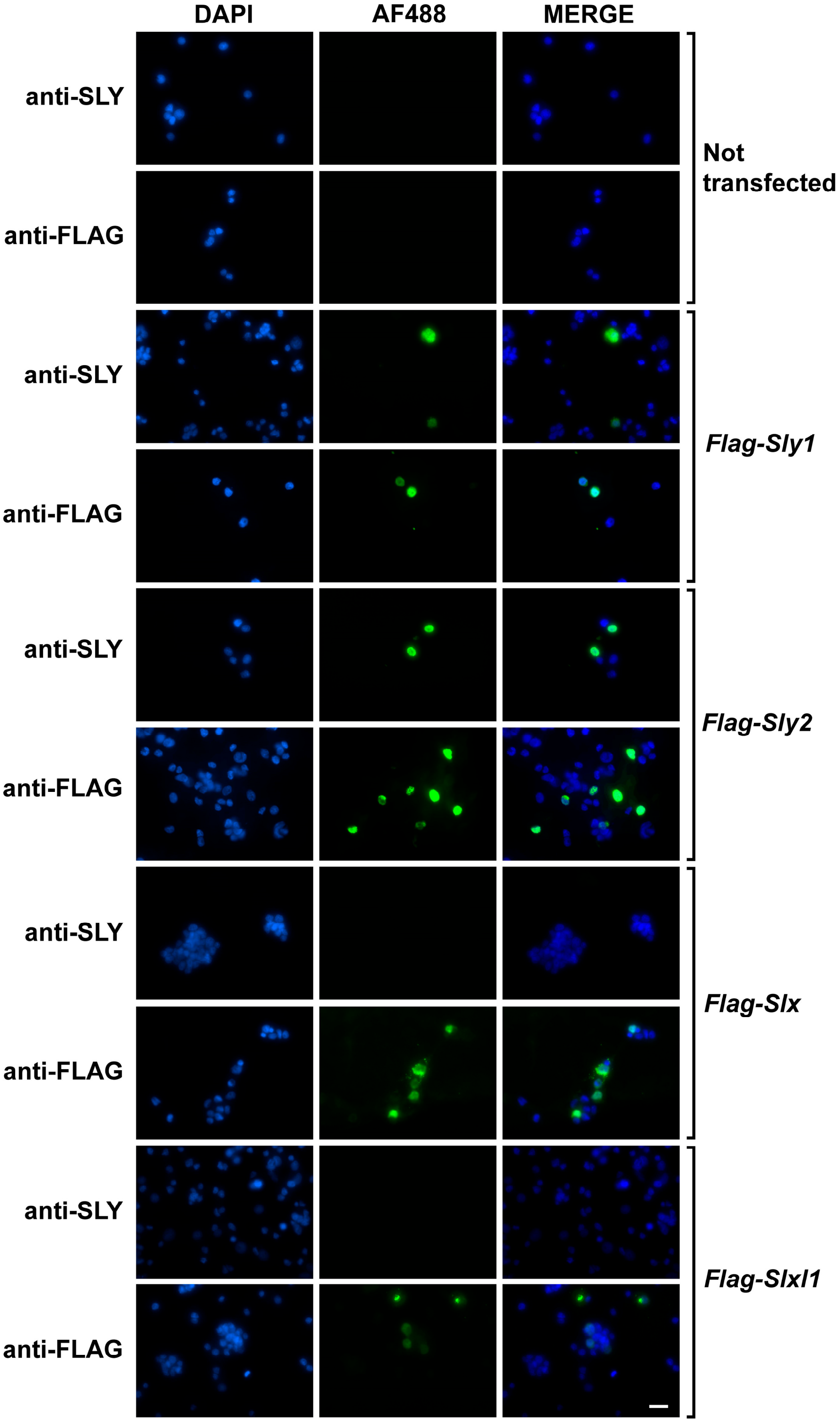

Figure S2. Characterization of anti-SLY antibody. Transfection of HEK296 cells with a *Flag-Sly1*, *Flag-Sly2*, *Flag-Slx*, and *Flag-Slxl1* constructs followed by immunostaining using an anti-FLAG antibody (green) and anti-SLY antibody (green). The cell nuclei were stained with DAPI (blue). Non-transfected cells served as negative control. Anti-FLAG antibody detected all fusion proteins while anti-SLY antibody detected only FLAG-SLY1 and FLAG-SLY2 fusion proteins. Bar = 100 μm.

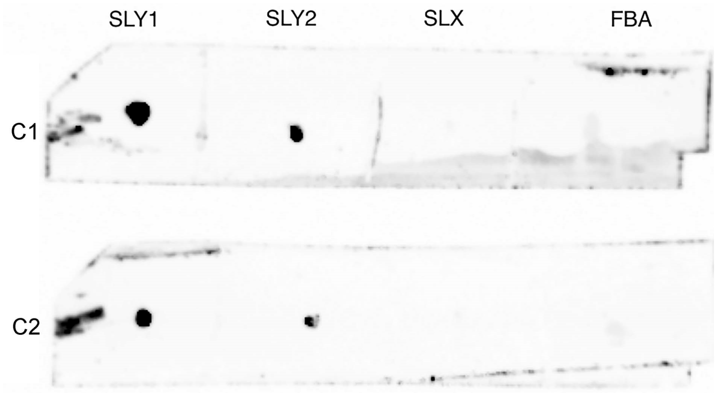

Figure S3. Dot-blot analysis. Detection of SLY1 and SLY2 proteins using anti-SLY antibody in membranes spotted with purified SLY1, SLY2, SLX and FBA proteins. Anti-SLY antibody detects SLY1 and SLY2 but not SLX and FBA. C1 and C2 represent two independent batches of anti-SLY proteins (i.e. two independent hybridoma culture supernatants).

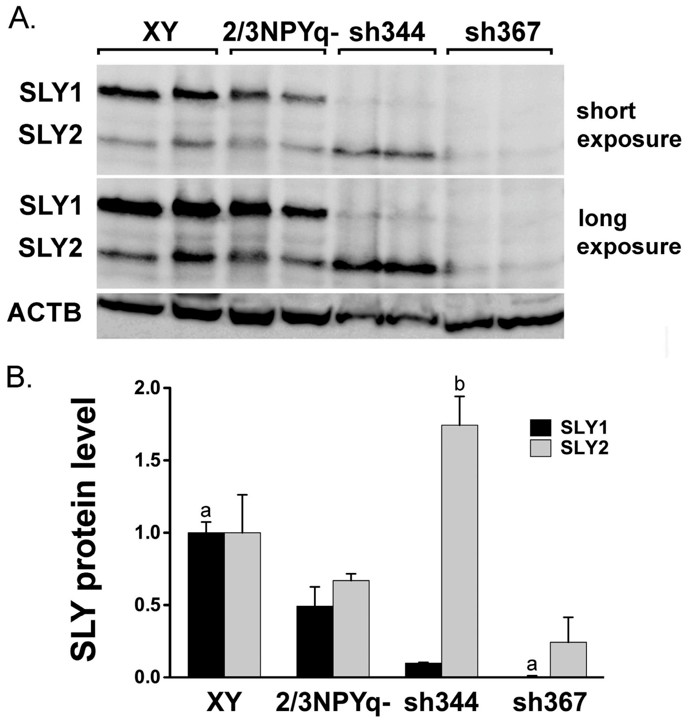

Figure S4. SLY expression in males with NPYq- and *Sly*-specific deficiency. (A) Exemplary western blot detection of SLY1 and SLY2 protein in testes from wild-type control (XY), mutant mice with a deletion removing 2/3 of the non-pairing Y chromosome long arm (2/3NPYq-) and Sly-KD transgenic mice with *Sly* deficiency (sh344 and sh367). (B) Levels of protein expression shown in panel A quantified with *ImageJ* software normalized with respect to ACTB signal and with XY data serving as normal expression baseline. The data represent an average ± SDev with n=2. Statistical significance (t-test): Comparison of genotypes for each protein isoform [a] different from all other; [b] different from 2/3NPYq- and sh367.

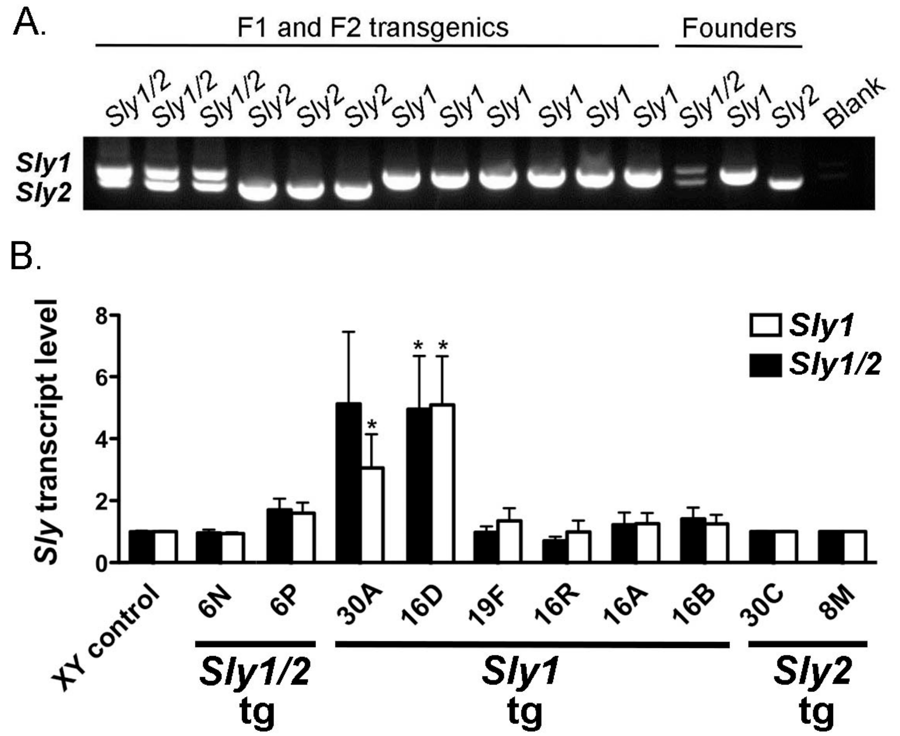

Figure S5. Production of mice transgenic for *Flag-Sly*. (A) Exemplary gel showing products of transgene-specific PCR amplifying *Sly1* and *Sly2* transgenes. F1 and F2 are offspring derived from transgenic founders. *Sly1*, *Sly2* and *Sly1/2* are mice carrying *Sly1*, *Sly2* and both *Sly1* and *Sly2* transgenes, respectively. (B) *Sly* transcripts levels (*Sly1* and *Sly1/2* global) in whole testes from F1 and F1 generation of *Sly* transgenic mice obtained by real-time RT-PCR with *Actb* as a loading control and negative siblings (XY control) as normal expression controls; there were no differences between negative siblings from different transgenic lines so the data from all of them were pooled. The graphs are mean ± SDev with number of males as follows: n=28 (XY), n=4 (30C), n=2 (8M) and n=5-12 (all other transgenic lines). Statistical significance (t-test, P<0.05): * different than respective transcript type in XY. Primer sequences are shown in Table S2.

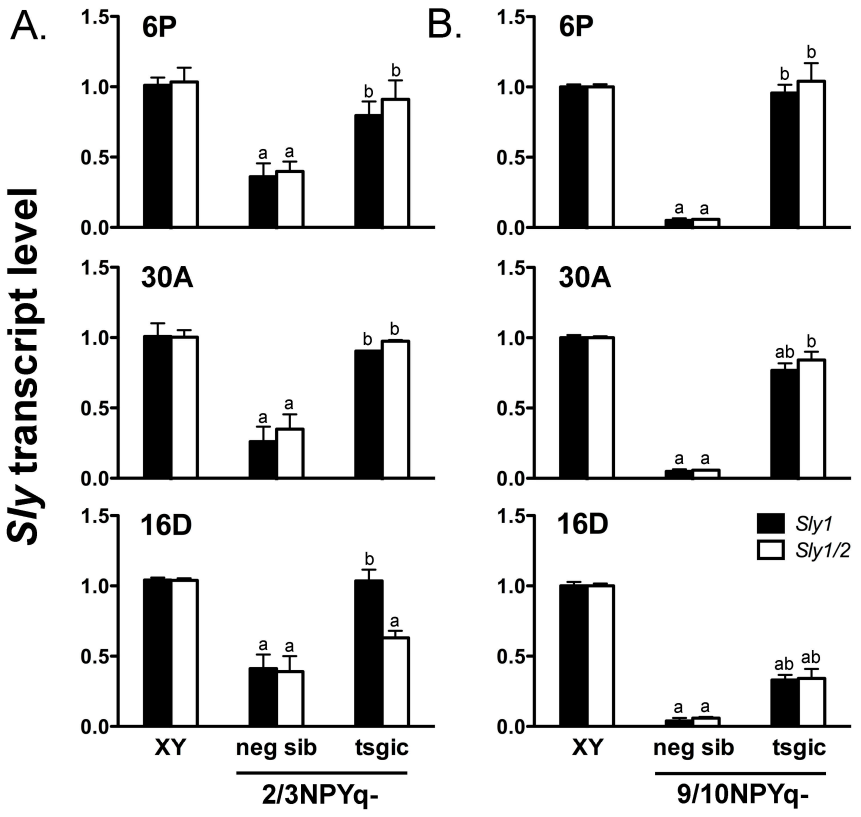

Figure S6. Addition of the *Flag-Sly* transgene to males with NPYq deletions rescues *Sly* expression deficiency. *Sly* transcripts levels (*Sly1* and *Sly1/2* global) in whole testes from moderately (A, 2/3NPYq-) and severely (B, 9/10NPYq-) NPYq deficient mice with (tsgic) and without (neg sib) *Flag-Sly* transgene addition obtained by real-time RT-PCR with *Actb* as a loading control and normalized to wild-type XY controls. Three transgenic lines were tested: 6P carrying *Sly1* and *Sly2* transgenes and lines 30A and 16D positive for *Sly1* transgene only. The graphs are mean ± SEM with n=3-9 (A) and n=3 (B). Statistical significance (t-test, P<0.05): [a] different than respective transcript type in XY; [b] different than respective transcript type in neg sib. Primer sequences are shown in Table S2. This figure is relevant to Fig. 3.

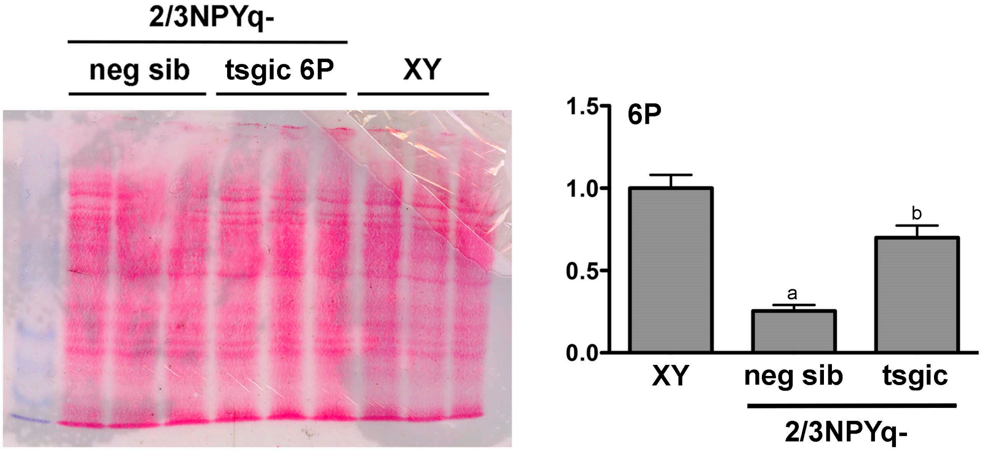

Figure S7. Addition of the *Flag-Sly* transgene to 2/3NPYq- males rescues SLY1 expression deficiency. Western blot was performed with whole testes lysates obtained from XY males and from males with moderate NPYq deficiency (2/3NPYq-) with (tsgic) and without (neg sib) *Flag-Sly* (line 6P) transgene addition. Levels of protein expression were quantified with *ImageJ* software and normalized to Ponceau signal. The data represent an average ± SEM with n=3. Statistical significance (t-test, $P<0.05$): [a] different from XY; [b] different from neg sib. This figure is relevant to Fig. 3 and shows the same data but after different normalization.

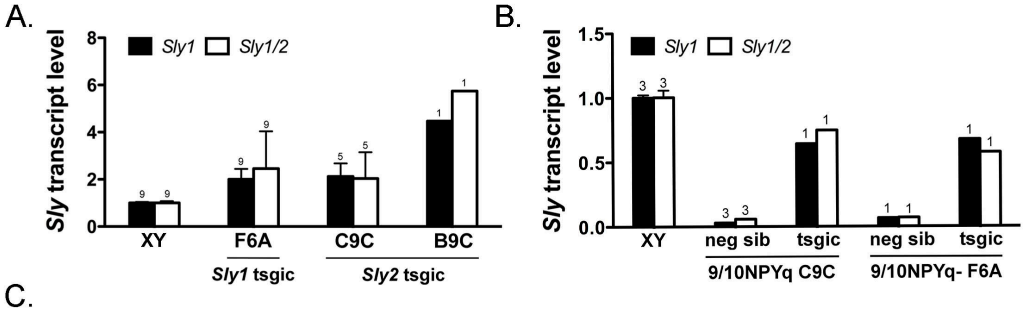

C.

Tg line	Genotype	No males	Age (weeks)	Average ± SEM			Eggs fertilized/eggs inseminated (%)
				Body weight (g)	Testis weight (mg)	Sperm number (1CE, x10[6])	
F6A	9/10NPYq-*Sly1*	1	9	28	63 ± 7	2.4	0/63 (0)
	9/10NPYq-	1	9	27	90 ± 3	1.1	0/85 (0)
B9C	9/10NPYq-*Sly2*	2	12	29 ± 3	75 ± 9	3.6 ± 4.4	0/162 (0)
	9/10NPYq-	3	12	33 ± 8	85 ± 16	3.1 ± 2.0	0/212 (0)
C9C	9/10NPYq-*Sly2*	7	12	27 ± 4	59 ± 14	1.2 ± 0.8	0/346 (0)
	9/10NPYq-	4	12	26 ± 5	56 ± 14	1.4 ± 2.0	0/104 (0)
WT	XY IVF control	5	15-27	34 ± 5	76 ± 8	5.6 ± 3.8	172/247 (70)

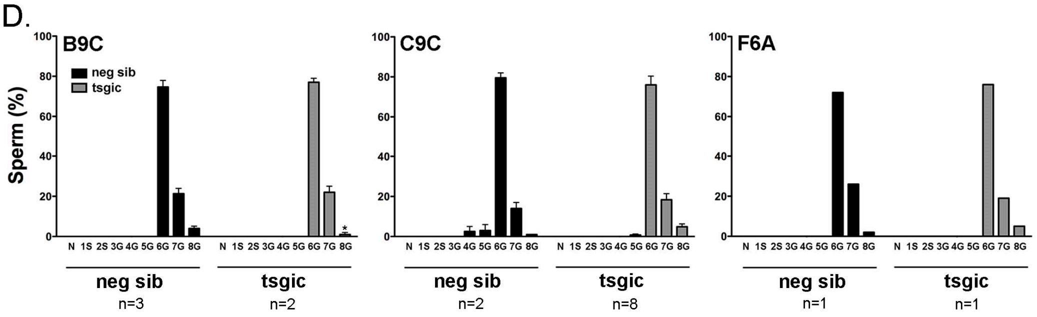

Figure S8. Addition of the *Sly* transgene (no FLAG tag) to males with severe NPYq deficiency rescues *Sly* expression but not low sperm number and sperm ability to fertilize oocytes in vitro. A & B: *Sly* transcripts levels (*Sly1* and *Sly1/2* global) in (A) whole testes from F1 and F1 generation of *Sly* transgenic mice and their negative siblings (XY) and (B) from 9/10NPYq deficient mice with (tsgic) and without (neg sib) *Sly* transgene addition obtained by real-time RT-PCR with *Actb* as a loading control and XY as normal expression controls; in A there were no differences between XY negative siblings from different transgenic lines so the data from all of them were pooled. The graphs are mean ± SEM with number of males shown above the individual bars. Primer sequences are shown in Table S2. C: Spermiogenic phenotype of 9/10NPYq deficient mice with (9/10NPYq-*Sly*) and without (9/10NPYq-) *Sly* transgene. 1CE = 1 cauda epididymis. D: Sperm headshape was evaluated in mice with 9/10NPYq- deficiency with (tsgic) and without (neg sib) the *Sly* transgenes. Three transgenic lines were tested: B9C and C9C positive for the *Sly2* transgene and F6A positive for the *Sly1* transgene. Normal headshape (N) and eight categories of headshape defects (slight: 1S-2S and gross: G3–G8) were differentiated. The graphs are average ± SDev with n shown under the graphs and 100 sperm examined per male. Statistical significance: two-way ANOVA with genotype and sperm headshape as factors revealed no effect of genotype ($P>0.05$), strong effect of headshape ($P<0.0001$), and no interaction effect ($P>0.05$) for all groups tested. The results of paired comparison for specific sperm headshape between transgenic and negative siblings in post-hoc Bonferroni test are shown within graphs: * $P<0.05$.

Table S1. Summary of mice used in this study.

Genotype	Mouse type	NPYq	Spermiogenic phenotype#
XY	wild-type	Intact	normal
2/3NPYq-	mutant	~2/3 deleted	moderate
9/10NPYq-	mutant	~9/10 deleted	severe
NPYq-	mutant	Lacking	severe
NPYq-2	mutant	Lacking	severe
sh344	transgenic	*Sly* knockdown	mild
sh367	transgenic	*Sly* knockdown	above moderate
XY*Sly1*	transgenic	*Sly1* overexpressed	normal*
XY*Sly2*	transgenic	*Sly2* overexpressed	normal*
XY*Sly1/2*	transgenic	*Sly1/2* overexpressed	normal*
2/3NPYq-*Sly1*	mutant transgenic	~2/3 deleted, *Sly1* overexpressed	moderate
2/3NPYq-*Sly1/2*	mutant transgenic	~2/3 deleted, *Sly1/2* overexpressed	moderate
9/10NPYq-*Sly1*	mutant transgenic	~9/10 deleted, *Sly1* overexpressed	severe
9/10NPYq-*Sly1/2*	mutant transgenic	~9/10 deleted, *Sly1/2* overexpressed	severe
NPYq-*Sly1*	mutant transgenic	lacking, *Sly1* expressed	severe

See Table 1 in Riel et al [12] for detailed spermiogenic phenotype summary for mutants and Sly-KD mice. * Only fertility was assessed.

Table S2. Primers.

Gene	Primer ID	Primer sequence	Reference
Genotyping Primers			
sh344	*sh344-F*	TAGCGCTACCGGACTCAGAT	[12]
	sh344-R	GTCCTCCTTGAAGTCGATGC	[12]
sh367	*sh367-F*	ACGTAAACGGCCACAAGTTC	[11]
	sh367-R	GTCCTCCTTGAAGTCGATGC	
Real-time PCR primers			
Sly1/2	*Sly Global-F*	CATTTATAAGACGCTTCACATAAAG	[11]
	Sly Global-R1	TCCTCCATGATGGCTCTTTC	
	Sly Global-R2	ATTCTCCATGATGGCTCTTTC	
Sly1	*Sly Long-F*	GAAGACATGGACATGAAGTAGG	[11]
	Sly Long-R1	Same as for *Sly* Global	
	Sly-Long-R2	Same as for *Sly* Global	
Actb	*Actb-F*	GGCACCACACCTTCTACAATG	[19]
	Actb-F	GTGGTGGTGAAGCTGTAGCC	
Acrv1	*Acrv1-F*	TGAGTACACCACTTCCAAGCA	[18]
	Acrv1-R	AAGCACATGTGTGGCAATTT	
Slx	*Slx-F*	TTCAGATGAAGAAGAAGAGCAGG	[13]
	Slx-R	TCCATATCAAACTTCTGCTCACAC	
Slx-like	*Slxl1-F*	TTGGAGGACGCTCATTCTG	[13]
	Slsl1-R	ACGACTTGTTGTTGATCATCTCC	
Actrt1	*Actrt1-F*	CTCAAAAATGGTCTGCAACAGC	[13]
	Actrt1-R	TCTTGATAGGGGTTCCCTCAAA	
Ssty1	*Ssty1-F*	AGAAGGATCCAGCTCTCTATGCT	[13]
	Ssty1-R	CCAGTTACCAATCAACACATCAC	
Ssty2	*Ssty2-F*	CAGGTGCCATTCTTACAGGACTAT	[13]
	Ssty2-R	ACCCAGGAACCTATTAAGAAGTCAT	
Asty	*Asty1-F*	GRGGAGTAGAACTCATCATC	[13]
	Asty1-R	CAGGAGATGACTAACATAGCA	
Ubb	*Ubb-F*	GAGGGGTGGCTATTAATTATTCG	[18]
	Ubb-R	CTAAACTTAAATTGGGGCAAGTG	
Mgclh	*Mgclh-F*	CCTTTACGTGTGACCTTTACCAG	[13]
	Mgclh-R	CTGAATATGACATTTCGGATATGGT	
Tnp1	*Tnp1-F*	TCAAGAGAGGTGGAAGCAAGA	[13]
	Tnp1-R	CACAAGTGGGATCGGTAATTG	
Prm1	*Prm1-F*	ACAAAATTCCACCTGCTCACA	[11]
	Prm1-R	GTTTTTCATCGGACGGTGGC	
Tcp11x2	*Tcp11x2k-F*	AAAGCCAATTCGTGGAGACAAT	[11]
	Tcp11x2-R	TGGGAGAGATGCAGAATATCCA	

Table S3. Relationship between SLY1/2 protein expression and spermiogenic phenotype and fertility of mice with NPY/*Sly* deficiency.

SLY1/2 global protein level	WT (100%) > sh344 Sly-KD (103%) > 2/3NPYq- (50%) > sh367 Sly-KD (12%) > 9/10NPYq- (1.3%) > NPYq- (0.7%)
Normal spermiogenesis & fertility[#]	WT > sh344 Sly-KD > 2/3NPYq- > sh367 Sly-KD > 9/10NPYq- > NPYq-2

n=7, 4, 6, 4, 4, 5 for WT, sh344, 2/NPYq-, sh367, 9.10NPYq-, NPYq-2. [#] See Table 1 in Riel et al [12] for detailed spermiogenic phenotype summary for mutants and Sly-KD mice. * Only fertility was assessed.
